# mTOR regulates brain morphogenesis by mediating GSK3 signaling

**DOI:** 10.1242/dev.108282

**Published:** 2014-11

**Authors:** Minhan Ka, Gianluigi Condorelli, James R. Woodgett, Woo-Yang Kim

**Affiliations:** 1 Developmental Neuroscience, Munroe-Meyer Institute, University of Nebraska Medical Center, Omaha, NE 68198, USA; 2 Humanitas Clinical and Research Center, University of Milan, Rozzano, Milan, Italy; 3 Samuel Lunenfeld Research Institute, Mount Sinai Hospital, Toronto M5G 1X5, Canada

**Keywords:** mTOR, GSK3, Neural progenitor, Neurogenesis, Neuron positioning, Mouse

## Abstract

Balanced control of neural progenitor maintenance and neuron production is crucial in establishing functional neural circuits during brain development, and abnormalities in this process are implicated in many neurological diseases. However, the regulatory mechanisms of neural progenitor homeostasis remain poorly understood. Here, we show that mammalian target of rapamycin (mTOR) is required for maintaining neural progenitor pools and plays a key role in mediating glycogen synthase kinase 3 (GSK3) signaling during brain development. First, we generated and characterized conditional mutant mice exhibiting deletion of mTOR in neural progenitors and neurons in the developing brain using Nestin-cre and Nex-cre lines, respectively. The elimination of mTOR resulted in abnormal cell cycle progression of neural progenitors in the developing brain and thereby disruption of progenitor self-renewal. Accordingly, production of intermediate progenitors and postmitotic neurons were markedly suppressed. Next, we discovered that GSK3, a master regulator of neural progenitors, interacts with mTOR and controls its activity in cortical progenitors. Finally, we found that inactivation of mTOR activity suppresses the abnormal proliferation of neural progenitors induced by GSK3 deletion. Our findings reveal that the interaction between mTOR and GSK3 signaling plays an essential role in dynamic homeostasis of neural progenitors during brain development.

## INTRODUCTION

Normal development of the brain, more than any other organ, requires precise control of the self-renewal and differentiation of neural progenitors to establish functional circuitry. Yet, the signaling network playing a decisive role in homeostatic control of balancing progenitors and neurons in the developing brain is poorly understood. The mammalian target of rapamycin (mTOR), a serine/threonine kinase, is essential for stem cell proliferation, growth and survival via protein synthesis and gene transcription (Hay and Sonenberg, 2004; Laplante and Sabatini, 2012; Ma and Blenis, 2009; Maiese et al., 2013). Recent studies suggest that mTOR signaling is involved in neural development (Hartman et al., 2013; Hentges et al., 1999, 2001; Rennebeck et al., 1998; Way et al., 2009). Neural roles of mTOR signaling have been increasingly pursued due to its association with neurodevelopmental disorders and brain cancers. Surprisingly, however, there is lack of *in vivo* genetic evidence that defines mTOR functions by deleting mTOR in neural progenitors and neurons during brain formation.

Previously, we have demonstrated that glycogen synthase kinase-3 (GSK3) is a master switch molecule for the regulation of neural progenitor self-renewal and neurogenesis during brain development (Kim et al., 2009). Still, little is known about GSK3 signaling mechanisms in neural progenitor regulation *in vivo*. The lack of this mechanistic knowledge sets a barrier in mapping out regulatory networks of neural progenitors and their progeny. Interestingly, GSK3 has been implicated in mTOR regulation. For example, recent studies reveal that GSK3 interacts with mTOR signaling in fibroblast cell lines via phosphorylation on TSC2, a component of mTOR repressor complex (Buller et al., 2008; Inoki et al., 2006). Furthermore, β-catenin signaling is negatively controlled by TSC in angiomyolipomas and lymphangioleiomyoma (Mak et al., 2005, 2003). Again, GSK3 modulates the TSC1/TSC2 complex via phosphorylation mechanisms in these cells (Mak et al., 2005). Thus, it is conceivable that the mTOR pathway plays a crucial role in transducing the GSK3 signal in neural cells of the developing brain.

Cortical neural progenitors in mice actively proliferate and generate neurons between embryonic day 10 and 17. *Mtor* null mice die around embryonic day 6 (Gangloff et al., 2004; Murakami et al., 2004), precluding the use of these mice in the analysis of mTOR functions in neural progenitors and their progeny. To investigate the functions and mechanisms of mTOR in the developing brain, we used conditional knockout strategies to target mTOR in neural cells. Conditional mTOR mutant mice generated a smaller brain with the decreases in the numbers of proliferating progenitors and cortical layer neurons. Abnormal cell cycle progression contributed to the disruption of neural progenitors in mTOR-deficient brains. The mTOR pathway is a downstream target of GSK3 signaling in neural progenitors. Our findings suggest that mTOR plays an important role in brain morphogenesis by regulating progenitors and their progeny.

## RESULTS

### mTOR deletion using a Nestin-cre driver decreases the size of the developing brain

**Fig. 1.**
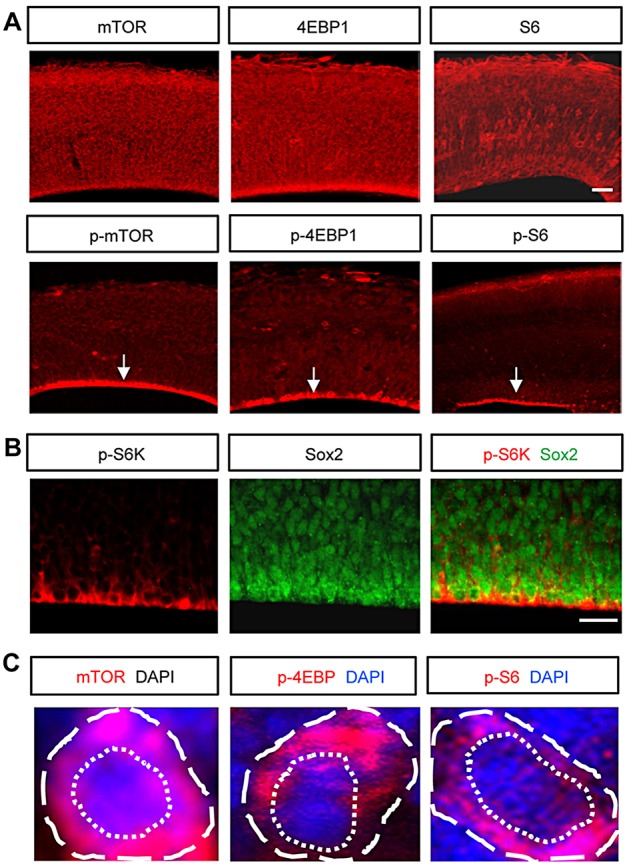
**Expression and activity of mTOR-signaling components in the developing brain.** (A) Top: mTOR signaling components were broadly expressed in the developing brain at E13.5. Bottom: phosphorylated forms of mTOR components were concentrated near the ventricular surface (arrows). Scale bar: 60 μm. (B) mTOR signaling was activated in Sox2-positive neural progenitors at the ventricular surface of E13.5 brain, as indicated by phospho-S6K expression. Scale bar: 20 μm. (C) Higher magnification images of cells immunostained with antibodies to mTOR, p-4EBP1 and p-S6. Dashed and dotted lines indicate a single cell and a nucleus, respectively.

We first assessed the expression patterns of mTOR-signaling components in the developing brain by using immunostaining. mTOR and its signaling molecules (i.e. 4EBP1 and S6) were broadly expressed in the cerebral cortex at E13.5 (Fig. 1A). These proteins are phosphorylated when the mTOR pathway is activated. Expression of phosphorylated forms of mTOR, 4EBP1, S6 and S6K were concentrated at the ventricular surfaces (Fig. 1A,B), indicating that the mTOR signal is activated in radial neural progenitors. Furthermore, ventricular labeling of phospho-S6K was associated with Sox2-positive radial neural progenitors (Fig. 1B). mTOR and phosphorylated targets were mostly distributed within the cytosolic areas (Fig. 1C). Together, these results suggest that mTOR activation may be important for neural progenitor development.

**Fig. 2.**
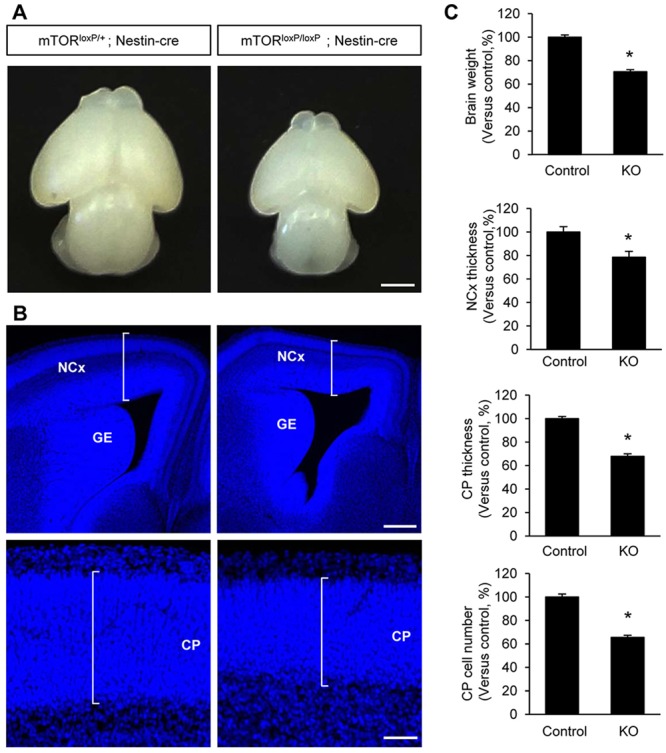
**mTOR deletion results in a smaller brain with a thinner cerebral cortex.** (A) Compared with control (*mTor^loxP/+^; Nestin-cre*) mice, *mTor^loxP/loxP^; Nestin-cre* embryos exhibit noticeably smaller brains at E15.5. Scale bar: 1 mm. (B) Coronal sections at E15.5 with DAPI staining shows marked reduction of the thicknesses of the neocortex and cortical plate in *mTor^loxP/loxP^; Nestin-cre* mice. Ventral brain regions, including ganglionic eminence, also appear to be reduced in the mutant brains. NCx, neocortex; CP, cortical plate; GE, ganglionic eminence. Scale bars: 500 μm. (C) Quantification of brain weight and of the thicknesses of the neocortex and cortical plate. Additionally, the numbers of cells in the cortical plate are assessed. Results are relative change versus control. Control is *mTor^loxP/+^; Nestin-cre*; knockout is *mTor^loxP/loxP^; Nestin-cre.* Data shown are mean±s.e.m. (*n*=20 sections from five animals for each condition). Significant difference when compared with controls (*P<0.05).

To test the role of mTOR in neural development, we examined mTOR deletion effects in the developing brain by generating conditional mTOR knockout mice (*mTor^loxP/loxP^; Nestin-cre*). mTOR floxed allele mice (Zhang et al., 2010) were crossed with a Nestin-cre line (Tronche et al., 1999). Western blots using brain lysates from E15.5 *mTor^loxP/loxP^; Nestin-cre* mice showed that mTOR is largely eliminated in the developing brain (supplementary material Fig. S1A,B). The levels of the phosphorylated targets of mTOR such as phospho-S6, phospho-S6K and phospho-4EBP1 were also markedly reduced in *mTor^loxP/loxP^; Nestin-cre* brain lysates, compared with control samples (supplementary material Fig. S1C,D). Furthermore, we assessed immunostaining patterns of mTOR, p-S6 and p-4EBP1 in E12.5 and E15.5 control and *mTor^loxP/loxP^; Nestin-cre* brains. At E12.5, mTOR was expressed in the VZ/SVZ and the cortical plate (supplementary material Fig. S2A,B). The phosphorylated targets p-S6 and p-4EBP1 were found in the VZ/SVZ. The fluorescence intensities of mTOR and its phosphorylated targets in these regions were reduced in *mTor^loxP/loxP^; Nestin-cre* brains. Importantly, the mTOR-deficient brains exhibit gross morphological abnormalities. Compared with the control littermate mice (*mTor^loxP/+^; Nestin-cre*), *mTor^loxP/loxP^; Nestin-cre* mice had smaller brains at E15.5 (Fig. 2A). *mTor^loxP/loxP^; Nestin-cre* brains weighed ∼30% less than control brains at E15.5 (Fig. 2C). Examining brain sections stained with DAPI revealed that *mTor^loxP/loxP^; Nestin-cre* brain exhibited thinner cerebral cortex and cortical plate throughout the rostrocaudal axis (Fig. 2B). *mTor^loxP/loxP^; Nestin-cre* brains also showed smaller ventral regions, including ganglionic eminence. Measurements of neocortex and cortical plate thicknesses showed 20-30% decrease in *mTor^loxP/loxP^; Nestin-cre* brains compared with control brains (Fig. 2C). The thinner thickness of cortical plate is a sign of reduced cell numbers. Indeed, there was a decrease of cortical plate cells by 40% in mTOR-deficient brains compared with controls (Fig. 2C). Cell density or size can contribute to the decrease in cortical thickness in the mutant brains. Thus, we examined cell density by counting DAPI-stained cells in the cortical plate. We found no difference in cell density between control and *mTor^loxP/loxP^; Nestin-cre* brains (supplementary material Fig. S3A,B). We also assessed cell size by labeling cortical cells using *in utero* electroporation of a GFP construct. There was a reduction in soma size in mTOR mutant cells compared with controls (supplementary material Fig. S3C,D). Thus, these experiments indicate that reduced cell size contributes to the phenotype of thinner cortical plate in mTOR mutant brains, as well as the abnormal neural progenitor proliferation. The brain phenotype of *mTor^loxP/loxP^; Nestin-cre* mice was not associated with cell death because there was no change in the level of cleaved caspase 3 in the mutant brain tissues (supplementary material Fig. S4).

### Elimination of mTOR suppresses proliferation of radial neural progenitors *in vivo*

**Fig. 3.**
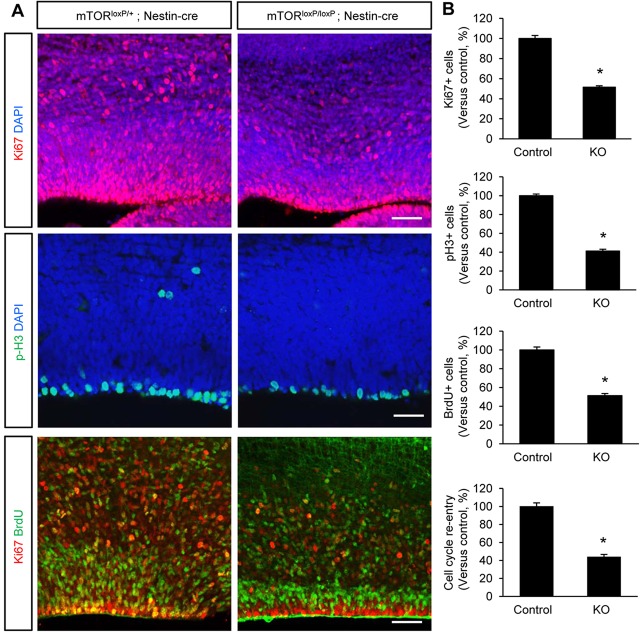
**Neural progenitor proliferation is suppressed in mTOR-deficient brain.** (A) Top and middle: brains from control and *mTor^loxP/loxP^; Nestin-cre* mice at E15.5 were immunostained with proliferation markers, anti-Ki67 or anti-phospho-histone H3 antibody. Neural progenitor proliferation at the ventricular zone was markedly reduced in *mTor^loxP/loxP^; Nestin-cre* brains. Bottom: cell cycle re-entry analysis was performed by treating E14.5 control and *mTor^loxP/loxP^; Nestin-cre* mice with BrdU for 24 h before brain harvest. Double immunostaining with BrdU (green) and Ki67 (red) antibodies was then performed. Cells that re-enter the cell cycle were reduced in mTOR mutant brains. Scale bars: 50 μm (top and bottom panels); 25 μm (middle panels). (B) Quantification of anti-phospho-histone H3-, Ki67- and BrdU-labeled cells in the ventricular and subventricular zones. The numbers of proliferating neurons to the DAPI-stained cells per field were counted and results are relative changes versus control. The index of cell cycle re-entry is defined as the fractions of both BrdU-positive and Ki67-positive cells in total BrdU-positive cells. Control is *mTor^loxP/+^; Nestin-cre*; knockout is *mTor^loxP/loxP^; Nestin-cre.* *P<0.05.

The smaller brain phenotype suggests that there is abnormal proliferation of neural progenitors in *mTor^loxP/loxP^; Nestin-cre* brains. To examine this possibility, we assessed the proportion of cells in various cell cycle phases at E15.5. We first started with Ki67 immunostaining, which generally labels actively proliferating cells at any stage. Ki67 staining showed that half as many cells were proliferating in mTOR-deficient brains compared with control brains (Fig. 3A,B). Then we assessed cells in mitotic phase by performing phospho-histone H3 immunostaining. Approximately 60% fewer progenitors at the ventricular zone were positive for phospho-histone H3 staining in *mTor^loxP/loxP^; Nestin-cre* brains compared with controls (Fig. 3A,B). We further assessed proliferating cells in S phase after a short term BrdU pulse. The number of BrdU-positive S phase cells was also decreased by 50% in *mTor^loxP/loxP^; Nestin-cre* brains (Fig. 3B).

To identify the underlying causes of the reduced progenitor pools, we investigated cell cycle re-entry by counting Ki67 and BrdU double-positive cells in total BrdU-positive cells after 24 h BrdU pulse. We found markedly less double-positive cells in *mTor^loxP/loxP^; Nestin-cre* brains (Fig. 3A,B), indicating decreased cell cycle re-entry. Additionally, we examined cell cycle length because prolonged cell cycle could also lead to a decrease in neural progenitor proliferation in mTOR-deficient brains. The numbers of Ki67 and BrdU double-positive cells in all Ki67-positive cells after a short BrdU pulse were reduced in *mTor^loxP/loxP^; Nestin-cre* brains (supplementary material Fig. S5), showing longer cell cycle in mTOR-deficient progenitors. Taken together, these results indicate that both a decrease in cell cycle re-entry and an increase in cell cycle length disrupt radial progenitor self-renewal, leading to the marked reduction in neural progenitor pools in *mTor^loxP/loxP^; Nestin-cre* mice.

### mTOR is required for efficient generation of intermediate neural progenitors

**Fig. 4.**
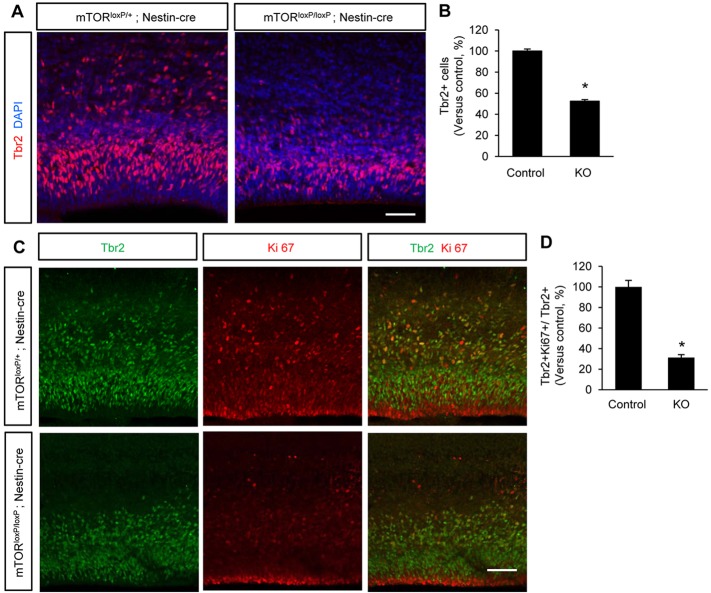
**Generation of intermediate neural progenitors is inhibited by mTOR deletion.** (A) The number of Tbr2-positive intermediate neural progenitors was markedly reduced in the *mTor^loxP/loxP^; Nestin-cre* brain sections. E15.5 cerebral cortex samples from controls and *mTor^loxP/loxP^; Nestin-cre* mice were immunostained with an anti-Tbr2 antibody. Scale bar: 50 μm. (B) Quantification of Tbr2-positive cells showed that compared with control, ∼50% fewer intermediate neural progenitors were generated in *mTor^loxP/loxP^; Nestin-cre* brains. Control is *mTor^loxP/+^; Nestin-cre*; knockout is *mTor^loxP/loxP^; Nestin-cre.* Data are relative change versus control. *P<0.05. (C) The number of proliferating progenitors was decreased in *mTor^loxP/loxP^; Nestin-cre* brains. E15.5 brain samples were co-immunostained with antibodies to Ki67 and Tbr2. Scale bar: 100 μm. (D) Quantification of C. Compared with control, the number of Ki67 and Tbr2 double-positive cells was decreased in *mTor^loxP/loxP^; Nestin-cre* brains. *P<0.05.

We wondered whether mTOR plays a role in the conversion of radial progenitors to intermediate progenitors. Thus, we immunostained cerebral cortex sections with an antibody to Tbr2, a marker for intermediate neural progenitors (Sessa et al., 2008). The number of Tbr2-positive cells was reduced by 50% in *mTor^loxP/loxP^; Nestin-cre* brains compared with control samples (Fig. 4A,B). Then, we assessed proliferating intermediate progenitors by co-immunostaining control and mTOR mutant brains with Tbr2 with Ki67 antibodies. We found that the number of Tbr2 and Ki67 double-positive cells out of Tbr2-positive cells was decreased by 67% in mTOR mutant brains compared with controls (Fig. 4C,D). These results show that mTOR plays a crucial role in producing and regulating intermediate progenitors during brain development.

### *mTor^loxP/loxP^; Nestin-cre* brains exhibit abnormal cortical neurogenesis

**Fig. 5.**
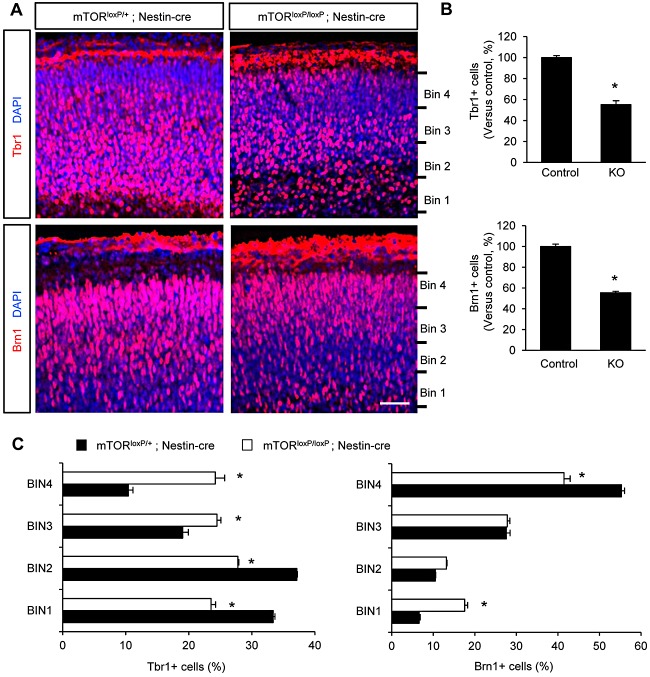
**mTOR deletion effects on cortical neurogenesis.** (A) Elimination of mTOR in neural progenitors resulted in fewer layer neurons in the developing brain. Brain sections from control and *mTor^loxP/loxP^; Nestin-cre* mice at E15.5 stage were immunostained by Tbr1 (top panels) and Brn1 (bottom panels). Both Tbr1- and Brn1-positive neurons were markedly less frequent in *mTor^loxP/loxP^; Nestin-cre* brain sections. Scale bar: 30 μm. (B) Quantification of Tbr1- and Brn1-positive cells. Control is *mTor^loxP/+^; Nestin-cre*; knockout is *mTor^loxP/loxP^; Nestin-cre.* *P<0.05. Data are mean±s.e.m. (C) Quantification of neuron positions in the cortical plates. Left: the graph indicates quantification of the distribution of Tbr1-positive neurons in the four bins dividing the thickness of the cortical plate as indicated in A in each genotype. Tbr1-positive neurons were distributed relatively evenly in the cortical plate of mTOR-deficient brains, whereas they were relatively accumulated in bin 1 and 2 in controls. Right: the distribution of Brn1-positive neurons was quantified. *P<0.05. Data are mean±s.e.m.

We assessed the number of cortical layer neurons in control and *mTor^loxP/loxP^; Nestin-cre* brains using immunostaining with antibodies to Tbr1 and Brn1 that label deeper layer and upper layer neurons, respectively. We found that Tbr1-positive neurons were decreased more than 40% in *mTor^loxP/loxP^; Nestin-cre* brains (Fig. 5A,B). Similarly, the numbers of Brn1-positive upper layer neurons were reduced in the mutant brains (Fig. 5A,B). These results demonstrate that mTOR is required for the generation of cortical neurons in developing brain. Together with the data presented in Fig. 3, these findings are consistent with the idea that the reduced neuron numbers in *mTor^loxP/loxP^; Nestin-cre* brains are caused by the defective maintenance of neural progenitor pools. Additionally, the positioning of cortical neurons was disrupted in *mTor^loxP/loxP^; Nestin-cre* brains. In control brain samples, Tbr1-positive neurons mostly localize within lower parts of the cortical plate (Fig. 5C). However, *mTor^loxP/loxP^; Nestin-cre* brain sections showed Tbr1 neurons located more evenly throughout the cortical plate. There was also abnormal localization of Brn1-positive neurons in the cortical plate. While Brn1-positive neurons were positioned mostly in the higher parts of the cortical plate in control brains, the distinct pattern was less obvious in *mTor^loxP/loxP^; Nestin-cre* brains (Fig. 5C).

We examined glial differentiation in mTOR-deleted brains, we measured the levels of GFAP (astrocyte) and Olig2 (oligodendrocyte) using western blotting. We found that the levels of GFAP and Olig2 were decreased by 41% and 53%, respectively, in mTOR mutant brains (supplementary material Fig. S6). These results suggest that mTOR is required for glial differentiation in the developing brain.

### mTOR deletion and radial neuron migration

**Fig. 6.**
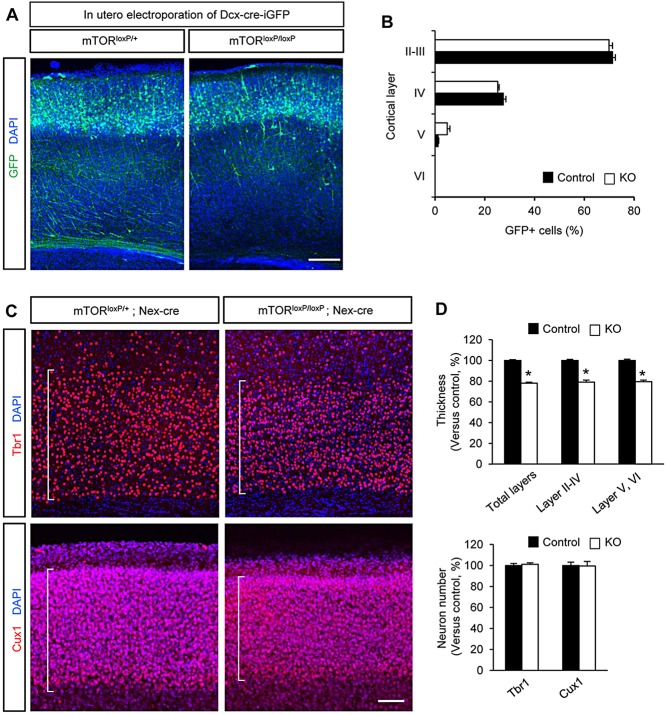
**The roles of mTOR in radial migration and growth of cortical neurons.** (A) mTOR deletion has no effect on radial migration of in developing cortical neurons. Control (*mTor^loxP/+^*) or *mTor^loxP/loxP^* embryos were electroporated *in utero* with Dcx-cre-iGFP at E13.5 to target radially migrating neurons. The electroporated brains were collected at P10 and neurons expressing GFP at the cortical plate were visualized. Scale bar: 200 μm. (B) Quantification of neuron positioning in the cortical plate. Control is *mTor^loxP/+^; Dcx-cre-iGFP*; knockout is *mTor^loxP/loxP^; Dcx-cre-iGFP.* Data are mean±s.e.m. (C) Effects of mTOR deletion in pyramidal neurons in the developing brain. Brain sections from P10 control (*mTor^loxP/+^; Nex-cre*) and *mTor^loxP/loxP^; Nex-cre* mice were immunostained with Tbr1 or Cux1 antibody. Cells were counterstained with DAPI. mTOR-deficiency in pyramidal neurons did not affect their positioning. Scale bar: 100 μm. (D) Quantification of the cortical thicknesses and neuron numbers in control and *mTor^loxP/loxP^; Nex-cre* cerebral cortex. The thicknesses of cortical layers containing Tbr1- and Cux1-positive neurons were decreased in mTOR-deleted mice. However, the numbers of neurons in the cortical layers were not statistically different, suggesting neuronal migration is independent of mTOR in the developing cortex. Control is *mTor^loxP/+^; Nex-cre*; knockout is *mTor^loxP/loxP^; Nex-cre.* *P<0.05. Data are mean±s.e.m.

Tracing newly born neurons after *in utero* electroporation of control and *mTor^loxP/loxP^; Nestin-cre* embryos with a GFP construct revealed abnormally distributed GFP-labeled neurons in the cerebral cortex (supplementary material Fig. S7A-C). These findings suggest a potential role of mTOR in neuronal migration during brain development. However, the defective neuronal positioning of *mTor^loxP/loxP^; Nestin-cre* brains could be induced indirectly by abnormal regulation of neural progenitors. Additionally, radial glial scaffold plays an important role in neuronal migration by providing paths for migrating neurons (Hatten, 1999; Marin et al., 2010). Indeed, *mTor^loxP/loxP^; Nestin-cre* brains had defective glial fibers, whereas control brain tissues showed robust glial processes (supplementary material Fig. S7D). These results suggest that neuronal positioning abnormalities in *mTor^loxP/loxP^; Nestin-cre* brains are partly due to the defective radial glial platforms. Thus, deleting mTOR using the Nestin-cre driver may be inconclusive in defining the role of mTOR in neuronal migration. To address this cell-autonomous issue, we deleted mTOR in developing neurons by performing *in utero* electroporation of *mTor^loxP/loxP^* mice with Dcx-cre-iGFP plasmid. This construct expresses Cre recombinase in only the neuronal population under the control of the Dcx promoter (Franco et al., 2011). The GFP domain is separated by IRES in the construct; thus, the Cre-expressing neurons can be marked and traced by GFP expression. After injecting Dcx-cre-iGFP into E13.5 control (*mTor^loxP/+^*) and *mTor^loxP/loxP^* mice, we analyzed the positioning patterns of radially migrating GFP-labeled neurons at P10. Surprisingly, we found that mTOR-deleted neurons showed no differences in migration patterns compared with control neurons (Fig. 6A,B). Most mTOR-deleted neurons migrated normally into the cortical plate in the developing cortex. Additionally, we used another strategy to delete mTOR in neurons *in vivo* using a Nex-cre mouse line (Goebbels et al., 2006; Wu et al., 2005). We examined Tbr1-positive deeper layer neurons and Cux1-positive upper layer neurons in P10 control (*mTor^loxP/+^; Nex-cre*) and *mTor^loxP/loxP^; Nex-cre* brains. Both Tbr1- and Cux1-positive neurons in *mTor^loxP/loxP^; Nex-cre* brains appeared to be localized within the deeper and upper layers, similar to those in controls (Fig. 6C). However, the thicknesses of cortical layers were decreased in *mTor^loxP/loxP^; Nex-cre* brains (Fig. 6C,D). In contrast to *mTor^loxP/loxP^; Nestin-cre* brains, examination of *mTor^loxP/loxP^; Nex-cre* samples revealed that the number of neurons in the cortical plate was not changed compared with controls (Fig. 6C,D). At E14.5, the thickness of Tbr1 layer and the number of Tbr1-positive cells were not altered in *mTor^loxP/loxP^; Nex-cre* brains (supplementary material Fig. S8).

### GSK3 controls the activity of mTOR signal

**Fig. 7.**
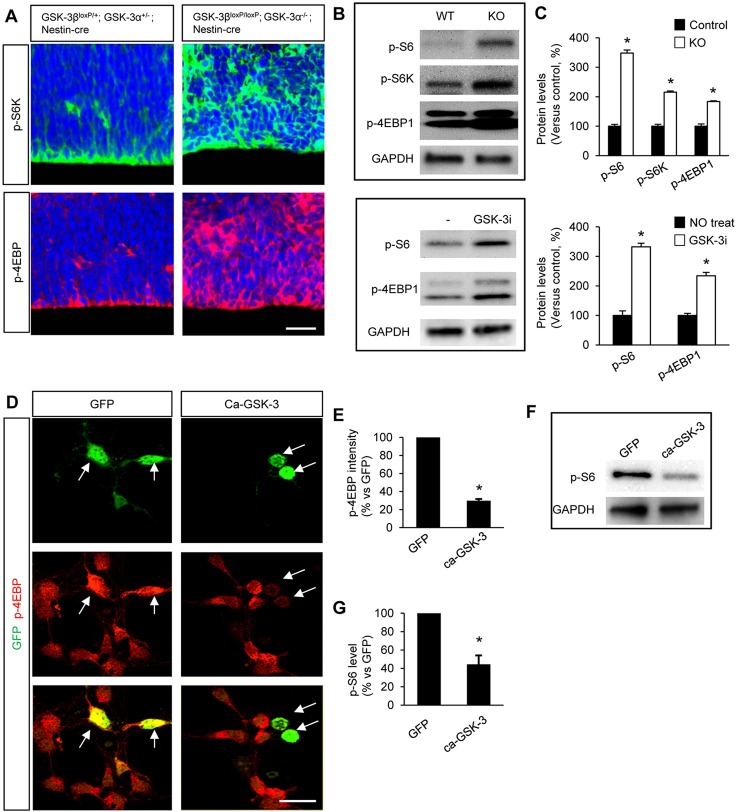
**mTOR activity is regulated by GSK3 in the developing neural progenitors.** (A) GSK3 elimination increases mTOR activity in the developing brain. mTOR activity was mostly localized in the ventricular zone in E13.5 control brains (*Gsk3*α*^+/−^; Gsk3*β*^loxP/+^; Nestin-cre*) as marked by phopho-S6K and phospho-4EBP1. In *Gsk3*α*^−/−^; Gsk3*β*^loxP/loxP^; Nestin-cre* brains, however, mTOR activity was expanded throughout the cerebral cortex. Scale bar: 25 μm. (B) Top: western blots at E13.5 showed that the levels of phospho-S6, phospho-S6K and phospho-4EBP1 were increased in GSK3-deficient brain tissues compared with the control. This indicates that GSK3 regulates mTOR activity in the developing brain. Control is *Gsk3*α*^+/−^; Gsk3*β*^loxP/+^; Nestin-cre*; knockout is *Gsk3*α*^−/−^; Gsk3*β*^loxP/loxP^; Nestin-cre.* Bottom: cortical progenitors from E13.5 mice were cultured in the presence or absence of GSK3 inhibitor lithium and then western blotting was performed using the cell lysates. Pharmacological inhibition of GSK3 increased mTOR activity and confirmed the *in vivo* results. (C) Quantification of western blots in B. Data are mean±s.e.m. *P<0.05. (D) Overexpression of GSK3 suppresses mTOR activity in cortical progenitors. Plasmids encoding a constitutively active GSK3β-GFP (ca-GSK3β-GFP) or GFP (control) were electroporated into E13.5 cortical neural progenitors. Cells were then cultured for 2 days and immunostained with phospho-4EBP1 antibody. Electroporated cells were identified by GFP expression (arrows). Cells expressing ca-GSK3β showed markedly reduced phospho-4EBP1 levels, whereas cells with control GFP expression had no effect. Scale bar: 20 μm. (E) The fluorescence intensities of phospho-4EBP1, as indicated in D, were quantified by ImageJ and are shown as relative changes versus control. *P<0.05. Data are mean±s.e.m. (F) Western blotting was performed using the lysates of the progenitors that were transfected with ca-GSK3β. The levels of phospho-S6 were reduced in cells expressing active GSK3β. (G) Quantification of phospho-S6 levels shown in F. *P<0.05. Data are mean±s.e.m.

The mTOR pathway interacts with GSK3 signaling (Inoki et al., 2006; Mak et al., 2005, 2003). We explore the potential roles of mTOR in GSK3 signaling during neural progenitor development, we first examined whether GSK3 controls the activity of mTOR signaling using GSK3-deleted brains. The mTOR activity was assessed by examining the expression levels and distribution patterns of phosphorylated forms of mTOR signaling components. In controls, phospho-S6K and phospho-4EBP1 were enriched along the ventricular surface (Fig. 7A). However, in *Gsk3*α*^−/−^; Gsk3*β*^loxP/loxP^; Nestin-cre* brains, phospho-S6K and phospho-4EBP1 were highly expressed throughout the developing cortex (Fig. 7A). Western blots revealed that the total levels of phospho-S6, phospho-S6K and phospho-4EBP1 were markedly increased in *Gsk3*α*^−/−^; Gsk3*β*^loxP/loxP^; Nestin-cre* brains (Fig. 7B, top; Fig. 7C, top). To confirm the effect of GSK3 on mTOR activity, we pharmacologically inhibited GSK3 activity in cultured progenitors. Western blots showed that pharmacological inhibition of GSK3 increased the levels of phospho-S6 and phospho-4EBP1 (Fig. 7B, bottom; Fig. 7C, bottom). These results show that mTOR signaling is markedly dysregulated in *Gsk3*α*^−/−^; Gsk3*β*^loxP/loxP^; Nestin-cre* neural progenitors, indicating that GSK3 controls mTOR activity in neural progenitors.

To further examine GSK3 roles in the control of mTOR activity, we overexpressed constitutively active GSK3 by transfecting ca-GSK3β-GFP (S9A) in dissociated cortical progenitors from E13.5 mice. Overexpression of a GFP plasmid (control) did not affect the phosphorylation levels of 4EBP1 (Fig. 7D,E). In cells overexpressing ca-GSK3β-GFP, however, the level of phospho-4EBP1 was decreased by 70% compared with control or non-transfected cells. Western blotting using an antibody to phospho-S6, another mTOR activity marker, confirmed the immunostaining results (Fig. 7F,G). Together, our results demonstrate that GSK3 controls the activity of mTOR signaling in neural progenitors during development.

### Inhibition of the mTOR signal suppresses hyperproliferation of GSK3-deleted neural progenitors

**Fig. 8.**
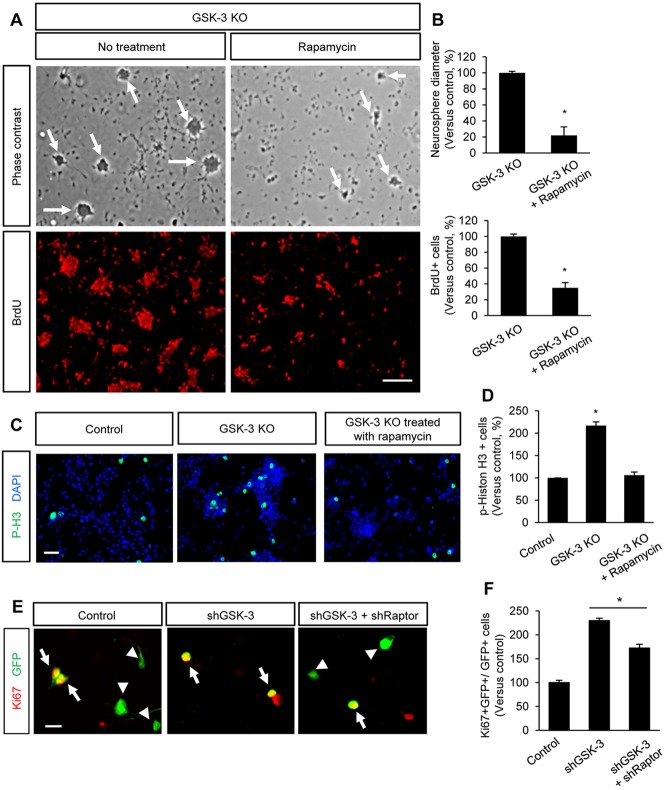
**Inhibition of mTOR suppresses proliferation of GSK3-deleted progenitors.** (A) Top: dissociated neural progenitors from E13.5 GSK3-deficient mice actively proliferated in culture and formed neurospheres (arrows). Inhibition of mTOR activity by treatment with rapamycin suppressed neurosphere formation in GSK3-deleted progenitor culture. Bottom: GSK3-deleted progenitors were cultured and treated with BrdU. Proliferating cells positive for BrdU were reduced by rapymycin treatment. Scale bar: 80 μm. (B) Neurosphere size was quantified in GSK3-deficient cortical progenitors in the absence or presence of rapamycin. The treatment of GSK3-deleted cells with rapamycin decreased the diameter of neurospheres and the number of BrdU-labeled cells. *P<0.05. Data are mean±s.e.m. (C) Compared with control cells, GSK3-deficient neural progenitors generated more mitotic cells (phospho-histone H3) due to their self-renewal capacity. However, when treated with rapamycin, their proliferation capacity was suppressed. Scale bar: 40 μm. (D) Quantification of phospho-histone H3-positive cells. Control is *Gsk3*α*^+/−^; Gsk3*β*^loxP/+^; Nestin-cre*; knockout is *Gsk3*α*^−/−^; Gsk3*β*^loxP/loxP^; Nestin-cre.* *P<0.05. Data are mean±s.e.m. (E) Progenitors were transfected with control GFP, shGSK3, or shGSK3 and shRaptor. Transfection of shRaptor suppressed the proliferation of GSK3-deleted cells. Arrows and arrowheads indicate proliferating and non-proliferating cells from a whole transfected cells, respectively. Scale bar: 20 μm. (F) Quantification of E. *P<0.05. Data are mean±s.e.m.

Next, we tested whether the changes of mTOR activity in GSK3-deleted progenitors are functionally relevant. We inhibited the mTOR function in dissociated *Gsk3*α*^−/−^; Gsk3*β*^loxP/loxP^; Nestin-cre* progenitors using the mTOR inhibitor rapamycin. When cultured, GSK3-deleted neural progenitors actively proliferated and formed neurospheres in less than 24 h (Fig. 8A). Control progenitors (*Gsk3*α*^+/−^; Gsk3*β*^loxP/+^; Nestin-cre*) did not form neurospheres in this time-frame (data not shown). Treatment of GSK3-deleted progenitors with rapamycin reduced the size and number of neurospheres (Fig. 8A,B). A BrdU proliferation assay also showed that mTOR inhibition decreased proliferation of GSK3-deleted neural progenitors (Fig. 8A,B). Furthermore, more mitotic cells, as indicated by phospho-histone H3 labeling, were found in GSK3-deleted progenitor culture compared with the control (Fig. 8C,D). Importantly, inhibition of the mTOR signal with rapamycin suppressed mitosis of GSK3-deleted progenitors. Additionally, we inhibited mTOR activity by transfecting an shRNA to Raptor, which is part of the mTOR complex 1, into cultured neural progenitors that simultaneously express an shGSK3 (Fig. 8E,F). shGSK3 overexpression increased progenitor proliferation, but co-expression shRaptor partially suppressed shGSK3-induced cell proliferation. These data reveal that the mTOR signaling is an important downstream target in GSK3 regulation of neural progenitors during development.

### TSC2 mediates the interaction between mTOR and GSK3

**Fig. 9.**
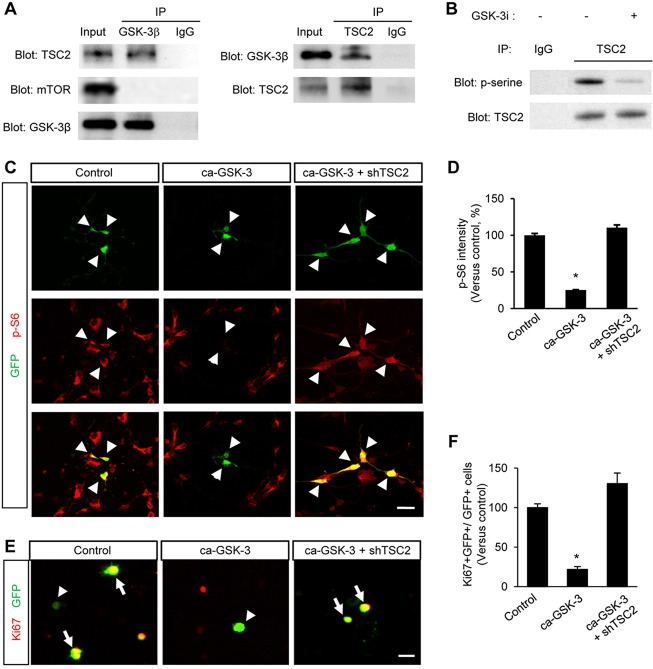
**TSC2 interaction with GSK3.** (A) GSK3 physically binds to TSC2. E13.5 brain lysates were immunoprecipitated with either GSK3β or TSC2 antibody, and subsequently subjected to western blotting. (B) GSK3 phosphorylates TSC2 *in vitro*. Brain lysates of E13.5 mice were immunoprecipitated using an anti-TSC2 antibody. The TSC2 fractions were then incubated with recombinant GSK3 and ATP in the presence or absence of a GSK3 inhibitor. Phosphorylated fractions of TSC2 were examined by western blotting using a phospho-serine antibody. Phosphorylation of TSC2 at serine residue(s) was found, whereas the level of phospho-serine was suppressed by a GSK3 inhibitor. (C) Elimination of TSC2 antagonizes the effects of GSK3 on mTOR activity. Cortical neural progenitors were cultured from E13.5 embryos and transfected with a constitutively active GSK3β plasmid (ca-GSK3β-GFP). The overexpression of ca-GSK3β-GFP marked by GFP labeling decreased phospho-S6 levels in neural progenitors. The phosphto-S6 levels, however, were rescued by co-overexpression of ca-GSK3β-GFP with an shTSC2 construct. Arrowheads indicate transfected cells. Scale bar: 20 μm. (D) The fluorescence intensity of phospho-S6 immunostaining shown in C was quantified by ImageJ. Data are presented as relative changes versus control±s.e.m. *P<0.05. (E) Elimination of TSC2 suppresses GSK3-induced inhibition of neural progenitor proliferation. E13.5 neural progenitors were cultured and transfected with control GFP, ca-GSK3, or ca-GSK3 and shTSC2. Proliferating neural progenitors were assessed by Ki67 immunostaining. Arrows and arrowheads indicate proliferating and non-proliferating cells out of a whole transfected cells, respectively. Scale bar: 20 μm. (F) The effects of GSK3, shTSC2 and shGSK3 on cell proliferation were assessed by quantifying the fraction of Ki67- and GFP-positive cells in total GFP-positive cells. *P<0.05. Data are mean±s.e.m.

TSC2 is an upstream negative regulator of mTOR and has potential GSK3 phosphorylation sites (Ma and Blenis, 2009), suggesting that GSK3 interacts with and phosphorylates TSC2. Thus, we first investigated whether GSK3 interacts with TSC2. E13.5 brain lysates were co-immunoprecipitated with GSK3β or TSC2 antibody, and subsequently immunoblotted with GSK3β, TSC2 and mTOR antibodies. We found that GSK3 was indeed physically bound to TSC2, but there was no direct binding of GSK3 with mTOR (Fig. 9A). This suggests that GSK3 interacts with TSC2 and this interaction regulates mTOR activity in neural progenitors. Next, we found that human recombinant GSK3 phosphorylates TSC2 at serine residue(s) *in vitro* (Fig. 9B). This result strongly suggests that endogenous GSK3 phosphorylates TSC2 in the developing brain.

Based on these results, we hypothesize that TSC2 mediates GSK-mediated control of mTOR activity. To test this idea, we transfected cultured neural progenitors with a control GFP, a ca-GSK3β-GFP, or a ca-GSK3β-GFP and an shTSC2 plasmid. Next, we assessed the mTOR activity using phospho-S6 immunostaining. Expression of a ca-GSK3β-GFP suppressed mTOR activity, whereas a control GFP expression had no effects (Fig. 9C,D). Importantly, expression of an shTSC2 abolished the negative effects of GSK3 overexpression on mTOR activity.

We also tested the functional relevance of TSC2 and GSK3 in neural progenitor proliferation. We cultured E13.5 cortical progenitors and transfected them as indicated in Fig. 9C. The elimination of endogenous TSC2 rescued the suppression of neural progenitor proliferation induced by GSK3 overexpression (Fig. 9E,F). Furthermore, we separately examined the rescue patterns of TSC2 knockdown in ca-GSK3-expressing progenitors of the ventricular zone or the subventricular/intermediate zones (supplementary material Fig. S9A). A recent study has shown that Pax6-positive apical progenitors are mostly localized in the ventricular zone, whereas a major part of Tbr2-positive intermediate progenitors is found in the subventricular and intermediate zones (Arai et al., 2011). We transfected either control GFP, ca-GSK3-GFP, or ca-GSK3-GFP and shTSC2 in neural progenitors and assessed proliferation patterns by immunostaining with a Ki67 and a GFP antibodies. We found that TSC2 knockdown suppressed the GSK3-mediated inhibition of progenitor proliferation in cells of both fractions (supplementary material Fig. S9B,C). Together, these findings suggest TSC2 as a mediator of mTOR/GSK3 signaling.

## DISCUSSION

Although studies using pharmacological approaches suggest mTOR functions in neural development, there is no *in vivo* genetic information about mTOR loss of function in the developing brain. Pharmacological manipulation often creates controversial outcomes (Lee et al., 2013; Ling et al., 2002; Pastalkova et al., 2006; Shema et al., 2007; Volk et al., 2013). Genetic elimination of mTOR suppresses expansion of neural progenitor pools, as indicated by decreases in brain size, numbers of cells that express mitotic markers and post-mitotic neurons in the cortical plates. Our data are consistent with the previous study that shows the role of TORC1 in neural stem cell regulation (Hartman et al., 2013). Knocking down mTORC1 activity in neonatal neural stem cells results in reduced expansion of neural stem cells and inhibits neurogenesis and differentiation (Hartman et al., 2013).

Cell cycle progression has been suggested as a key control point for mechanisms that account for neuron numbers in the brain (Dehay and Kennedy, 2007). Cell-cycle re-entry/exit is an indication of cell cycle progress (Chenn and Walsh, 2002; Katayama et al., 2011; Komada et al., 2008). mTOR-deficient progenitors show a failure of cell cycle re-entry. Another factor that influences neural progenitor self-renewal is the speed of the cell cycle. Changes in cell-cycle length have previously been implicated in altered cell-cycle progression (Dehay and Kennedy, 2007; Kim et al., 2009; Qu et al., 2013). Importantly, the mTOR signal is highly associated with cell-cycle progression in several cell types, and inhibition of mTOR pathway using a pharmacological inhibitor, rapamycin, induces cell-cycle arrest (Rosner et al., 2009; Song et al., 2007; Wanner et al., 2006). Consistently, our data show that elimination of mTOR results in slow cell-cycle rate. Thus, mTOR-deficient neural progenitors appear to have cell-cycle arrest and subsequently exit from the cell cycle.

We have observed that mTOR is expressed in the cortical plate where neuroblasts exist, as well as in the VZ/SVZ at early developmental stage. Crossing *mTOR-floxed* allele with *Nestin-cre* driver decreased the intensity of immunostained mTOR in both the cortical plate and the VZ/SVZ. The fluorescence intensities of immunostained targets are reduced in *mTor^loxP/loxP^; Nestin-cre* brains. We measured the intensity of mTOR immunostaining to assess the degree of mTOR recombination in the brain. The intensity measurement would not necessarily reflect the recombination of mTOR gene. More importantly, a recent report has shown that the Nestin-cre line used in our study is insufficient for recombination in the cortical VZ and SVZ at early stages such as E12.5 (Liang et al., 2012). Minimal recombination in the VZ and SVZ of the Nestin-cre line happens at E12.5. Thus, these findings suggest that early phenotypes of mTOR brain up until E14.5 are caused by postmitotic neuroblasts and immature neurons, which impact VZ/SVZ progenitors non-autonomously.

Cell density and size are factors that can determine the thickness of the cortical plate. Furthermore, changes in mTOR signaling are associated with changes in cell size (Tsai et al., 2014). Whereas *mTor^loxP/loxP^; Nestin-cre* brain tissues show no difference in cell density, they have smaller cells in the cortical plate. Thus, reduced cell size contributes to the thinner cortical plate in *mTor^loxP/loxP^; Nestin-cre* brains, as well as to the abnormal neural progenitor proliferation. Similar to the decrease of dividing radial neural progenitors, the population of intermediate neural progenitors marked with Tbr2 is markedly downregulated in the absence of mTOR. The decreased numbers of both post-mitotic neurons and intermediate progenitors in mTOR-deficient mice are expected because radial neural progenitors are the source of both cell types. Thus, neural differentiation is largely arrested at the radial progenitor stage in mTOR-deficient brain.

We have previously demonstrated the requirement of GSK3 in neural progenitor self-renewal and neurogenesis *in vivo* (Kim et al., 2009). However, the GSK3 signaling mechanisms underlying the coordinated temporal and spatial control of neural progenitors have remained unclear. Recent studies suggest that GSK3 directly phosphorylates TSC2, a component of mTOR repressor complex, in fibroblast cell lines; this phosphorylation modulates mTOR activity (Buller et al., 2008; Inoki et al., 2006; van Diepen et al., 2009). Furthermore, TSC negatively regulates β-catenin signaling that is mainly controlled by GSK3 in cancer cells (Mak et al., 2005, 2003). As in fibroblasts, GSK3 modulates TSC1/TSC2 complex via phosphorylation mechanisms in cancer cells (Mak et al., 2005). Consistently, our results have demonstrated that GSK3 controls mTOR activity in developing neural progenitors. Genetic or pharmacological inhibition of GSK3 leads to elevation of mTOR activity in neural progenitors. By contrast, overexpression of GSK3 suppresses mTOR activity. Importantly, we have found that inhibition of mTOR signal suppresses the hyperproliferation of GSK3-deficient neural progenitors. This regulation of mTOR activity by GSK3 appears to be mediated by TSC2 because GSK3 directly binds to and phosphorylates TSC2 in neural progenitors. Furthermore, co-expression of shRNA to TSC2 reverses the GSK3 overexpression effects on mTOR activity. The amino acid(s) on which GSK3 phosphorylates TSC2 in neural progenitors remain to be elucidated in the future. Our findings together indicate that mTOR mediates GSK3 signaling to control neural progenitor proliferation. The brain phenotypes of GSK3 double knockout mice are described in our previous publication (Kim et al., 2009). GSK3 double-knockout brains show greater degree of progenitor pool expansion than mTOR knockout brains, suggesting GSK3 uses not only mTOR but other signaling pathways such as β-catenin and SHH pathways (Kim et al., 2009).

Extracellular factors and intracellular molecules, including BDNF, IGF, ERB, AKT, PI3K and GSK3 crucially control cortical neuron migration. mTOR is part of the signaling pathways containing all these components, suggesting that mTOR plays a role in neuron migration. Indeed, a recent study reported that RTP801/REDD1, an inhibitor of mTOR activation, is associated with cortical neuron localization (Malagelada et al., 2011). Surprisingly, there are no defective patterns of cortical layering in *mTor^loxP/loxP^; Nex-cre* mice. Brain size and cortical layer thickness are reduced in *mTor^loxP/loxP^; Nex-cre* mice compared with controls, but neuronal numbers of the layers are not changed. Furthermore, these mice survive at postnatal stages. Migration of GFP-positive neurons after Dcx-cre-iGFP injection into *mTor^loxP/loxP^* brain confirms no effect of mTOR deletion, at least in radial neuron migration in the cerebral cortex. Our data are consistent with the previous observation that, whereas AKT and PI3K are required for cortical neuron migration, the mTOR/S6K pathway is dispensable (Segarra et al., 2006). Thus, although mTOR is an important downstream mediator of growth factor/AKT/PI3K pathways, mTOR does not seem to participate in the pathways controlling radial migration of cortical projection neurons. However, mTOR fluorescence intensity within the VZ and SVZ of *mTor^loxP/loxP^; Nex-cre* mice was not significantly different from that of wild-type controls at the early stage, E14.5. Furthermore, mTOR immunostaining intensity in the cortical plate also appeared to be not changed at this age. Accordingly, the thickness of Tbr1-positive cells in the cerebral cortex was not changed in E14.5 *mTor^loxP/loxP^; Nex-cre* mice compared with control mice. Therefore, mTOR deletion may not be sufficient during early stage migration and might become more robust at later stages. The reduced thickness of Tbr1 and Cux1 layers in P10 *mTor^loxP/loxP^; Nex-cre* mice may reflect the potential effects of mTOR in neuronal migration and final positioning. Although Dcx-cre-mediated deletion of mTOR did not show migration abnormality in the cerebral cortex, we cannot exclude the possibility that mTOR has a role for neuronal migration and subsequent positioning in the developing brain.

## MATERIALS AND METHODS

### Materials

Rapamycin (Cell Signaling), GSK3 inhibitor (Calbiochem), human FGF (R&D system), human EGF (R&D system), B27 supplements (Invitrogen), N2 supplement (Invitrogen) and BrdU (Sigma) were purchased from the companies indicated.

Dcx-cre-iGFP plasmid was generously provided by Dr Ulrich Mueller (The Scripps Research Institute, La Jolla, CA, USA). To generate shTSC2, we targeted a sequence (5′-TGAACATAGGACCATG-3′) and its complement, and then cloned them into a modified pSuper-Basic vector, as previously described (Kim et al., 2006). A sequence (5′-CGTGCTAACAGCATTAA-3′) and its complement were used to make shRaptor construct. Constitutively active GSK3β (S9A) and shGSK3 have been described in previous papers (Kim et al., 2006; Stambolic and Woodgett, 1994).

### Mice

Mice were handled according to our animal protocol approved by the University of Nebraska Medical Center. mTOR floxed mice (Zhang et al., 2010), GSK3α null mice (MacAulay et al., 2007) and GSK3β floxed mice (Patel et al., 2008) have been described previously. Nestin-cre (Tronche et al., 1999) or Nex-cre mice (Goebbels et al., 2006) were used to generate conditional mTOR and GSK3 knockout mice (*mTor^loxP/loxP^; Nestin-cre*, *mTor^loxP/loxP^; Nex-cre*, and *GSK3α^−/−^ GSK3β^loxP/loxP^; Nestin-cre*). The Nex-cre mouse was generously provided by Dr Klaus-Armin Nave (Max Planck Institute, Göttingen, Germany).

### Immunohistochemistry

Immunohistochemical labeling of embryonic brain sections or dissociated neural cells was performed as described previously (Kim et al., 2006). The following primary antibodies were used: rabbit anti-mTOR (Cell Signaling), rabbit anti-4EBP1 (Cell Signaling), mouse anti-S6 (Cell Signaling), rabbit anti-TSC2 (Cell Signaling), rabbit anti-phospho-mTOR (Cell Signaling), rabbit anti-phospho-4EBP1 (Cell Signaling), rabbit anti-phospho-S6 (Cell Signaling), rabbit anti-phospho-S6K (Cell Signaling), rabbit anti-phospho-histone H3 (Upstate Biotech), mouse anti-BrdU (Sigma), rabbit anti-Ki67 (Covance), rabbit anti-Sox2 (Chemicon), rabbit anti-Tbr1 (Chemicon), rabbit anti-Cux1 (Santa Cruz), goat anti-Brn1 (Novus Biologicals), rabbit anti-Tbr2 (Abcam), mouse anti-Tuj1 (Sigma). Appropriate secondary antibodies conjugated with Alexa Fluor dyes (Invitrogen) were used to detect primary antibodies.

### *In utero* electroporation

Timed pregnant female mice from E13.5 day of gestation were deeply anesthetized and the uterine horns were gently exposed. The lateral ventricles of an embryonic brain were injected with plasmid DNA (2 μg/μl). Electroporation was achieved by placing two sterile electrodes on opposing sides of the uterine sac around the embryonic head and applying a series of short electrical pulses (five pulses separated by 900 ms were applied at 40 V). The small electrical pulses drive charged DNA constructs into surrounding cells in the embryonic brain. Embryos were allowed to develop *in utero* for the indicated time.

### Morphometry

For the quantification of cortical plate thickness, images of 20 different brain sections at periodic distances along the rostrocaudal axis were taken with Zeiss LSM510 and LSM710 confocal microscopes and a Nikon Eclipse epifluorescence microscope attached to a QImaging CCD camera. The images were analyzed by using ZEN (Zeiss), LSM image browser (Zeiss), QCapture software (QImaging) and ImageJ (NIH). The calculated values were averaged and some results were recalculated as relative changes versus control.

For cell counts, numbers of neural progenitors and neurons positive to BrdU, phospho-histone H3, Ki67, Tuj1, Tbr1, Cux1, Brn1, Tbr2 or DAPI were obtained as described previously (Cappello et al., 2006; Chenn and Walsh, 2002). Ten mice for each experiment (control mice, *n*=5; mutant mice, *n*=5) were used. More than 20 coronal tissue sections alongside the rostrocaudal axis from each embryonic brain were examined. For analyzing cultured cells, more than 20 fields were analyzed in each condition (*n*=100).

### BrdU administration and cell cycle analysis

For proliferation assays, intraperitoneal injection was performed into pregnant mice at E13.5-15.5. BrdU (20 mg per kg body weight, dissolved in 0.9% saline) was administered before sacrifice. For the analysis of cell cycle re-entry, control and *mTor^loxP/loxP^; Nestin-cre* mice were exposed to BrdU for 24 h. Brain slices were then immunostained with antibodies to BrdU and Ki67. The ratio of cells labeled with BrdU and Ki67 to total cells that incorporated BrdU was determined. For the analysis of cell cycle length, the ratio of progenitor cells positive for Ki67 and BrdU to the total Ki67 labeled cells was assessed after a 1 h BrdU pulse.

### Western blotting

Lysates from E13.5 telencephalon were prepared using RIPA buffer and the protein content was determined by a Bio-Rad Protein Assay system. Primary antibodies: rabbit anti-phospho-S6 (Cell Signaling), rabbit anti-phospho-S6K (Cell Signaling), rabbit anti-phospho-4EBP1 (Cell Signaling), rabbit anti-GAPDH (Cell Signaling), rabbit anti-TSC2 (Cell Signaling), rabbit anti-mTOR (Cell Signaling), rabbit anti-phospho-serine (Abcam), mouse anti-GSK3β (BD Transduction Laboratory) or rabbit anti-GSK3β antibody (Upstate). Appropriate secondary antibodies conjugated to HRP were used (Cell Signaling) and the ECL reagents (Amersham) were used for immunodetection.

For quantification of band intensity, blots from three independent experiments for each molecule of interest were used. Signals were measured using ImageJ software and represented by relative intensity versus control. GAPDH was used as an internal control to normalize band intensity.

### Progenitor cultures

Cerebral cortex from E12.5-14.5 mice was isolated and dissociated with trituration after trypsin/EDTA treatment. The cells were then plated onto poly-D-lysine/laminin-coated coverslips and cultured for 2 days in the medium containing neurobasal medium, B27 and N2 supplements, FGF (10 ng/ml), and EGF (10 ng/ml). For differentiation of neural progenitors, the medium containing FGF and EGF was removed and replaced by neurobasal medium with 5% serum, B27 and N2 supplements.

### Cell transfection

Mouse cortical progenitors were transfected with various plasmids as described previously (Kim et al., 2006). Briefly, embryonic cortices were dissociated and suspended in 100 ml of Amaxa electroporation buffer with 1-10 μg of plasmid DNA. Suspended cells were then transferred to Amaxa electroporation cuvette and electroporated with an Amaxa Nucleofector apparatus.

### Statistical analysis

Statistical significance was determined with independent *t*-test for two-population comparison and one way analysis of variance followed by Tukey's post-hoc test for multiple comparisons. Unless otherwise noted, data are presented as mean±s.e.m. The test was considered significant when *P*<0.05.

## Supplementary Material

Supplementary Material

## References

[DEV108282C1] Arai Y., Pulvers J. N., Haffner C., Schilling B., Nüsslein I., Calegari F. and Huttner W. B. (2011). Neural stem and progenitor cells shorten S-phase on commitment to neuron production. *Nat. Commun.* 2, 154 10.1038/ncomms1155 21224845 PMC3105305

[DEV108282C2] Buller C. L., Loberg R. D., Fan M.-H., Zhu Q., Park J. L., Vesely E., Inoki K., Guan K.-L. and Brosius F. C. III (2008). A GSK-3/TSC2/mTOR pathway regulates glucose uptake and GLUT1 glucose transporter expression. *Am. J. Physiol. Cell Physiol.* 295, C836-C843 10.1152/ajpcell.00554.2007 18650261 PMC2544442

[DEV108282C3] Cappello S., Attardo A., Wu X., Iwasato T., Itohara S., Wilsch-Bräuninger M., Eilken H. M., Rieger M. A., Schroeder T. T., Huttner W. B. et al. (2006). The Rho-GTPase cdc42 regulates neural progenitor fate at the apical surface. *Nat. Neurosci.* 9, 1099-1107 10.1038/nn1744 16892058

[DEV108282C4] Chenn A. and Walsh C. A. (2002). Regulation of cerebral cortical size by control of cell cycle exit in neural precursors. *Science* 297, 365-369 10.1126/science.1074192 12130776

[DEV108282C5] Dehay C. and Kennedy H. (2007). Cell-cycle control and cortical development. *Nat. Rev. Neurosci.* 8, 438-450 10.1038/nrn2097 17514197

[DEV108282C6] Franco S. J., Martinez-Garay I., Gil-Sanz C., Harkins-Perry S. R. and Müller U. (2011). Reelin regulates cadherin function via Dab1/Rap1 to control neuronal migration and lamination in the neocortex. *Neuron* 69, 482-497 10.1016/j.neuron.2011.01.003 21315259 PMC3056352

[DEV108282C7] Gangloff Y.-G., Mueller M., Dann S. G., Svoboda P., Sticker M., Spetz J.-F., Um S. H., Brown E. J., Cereghini S., Thomas G. et al. (2004). Disruption of the mouse mTOR gene leads to early postimplantation lethality and prohibits embryonic stem cell development. *Mol. Cell. Biol.* 24, 9508-9516 10.1128/MCB.24.21.9508-9516.2004 15485918 PMC522282

[DEV108282C8] Goebbels S., Bormuth I., Bode U., Hermanson O., Schwab M. H. and Nave K.-A. (2006). Genetic targeting of principal neurons in neocortex and hippocampus of NEX-Cre mice. *Genesis* 44, 611-621 10.1002/dvg.20256 17146780

[DEV108282C9] Hartman N. W., Lin T. V., Zhang L., Paquelet G. E., Feliciano D. M. and Bordey A. (2013). mTORC1 targets the translational repressor 4E-BP2, but not S6 kinase 1/2, to regulate neural stem cell self-renewal in vivo. *Cell Rep.* 5, 433-444 10.1016/j.celrep.2013.09.017 24139800

[DEV108282C10] Hatten M. E. (1999). Central nervous system neuronal migration. *Annu. Rev. Neurosci.* 22, 511-539 10.1146/annurev.neuro.22.1.511 10202547

[DEV108282C11] Hay N. and Sonenberg N. (2004). Upstream and downstream of mTOR. *Genes Dev.* 18, 1926-1945 10.1101/gad.1212704 15314020

[DEV108282C12] Hentges K., Thompson K. and Peterson A. (1999). The flat-top gene is required for the expansion and regionalization of the telencephalic primordium. *Development* 126, 1601-1609. 10079223 10.1242/dev.126.8.1601

[DEV108282C13] Hentges K. E., Sirry B., Gingeras A.-C., Sarbassov D., Sonenberg N., Sabatini D. and Peterson A. S. (2001). FRAP/mTOR is required for proliferation and patterning during embryonic development in the mouse. *Proc. Natl. Acad. Sci. USA* 98, 13796-13801 10.1073/pnas.241184198 11707573 PMC61121

[DEV108282C14] Inoki K., Ouyang H., Zhu T., Lindvall C., Wang Y., Zhang X., Yang Q., Bennett C., Harada Y., Stankunas K. et al. (2006). TSC2 integrates Wnt and energy signals via a coordinated phosphorylation by AMPK and GSK3 to regulate cell growth. *Cell* 126, 955-968 10.1016/j.cell.2006.06.055 16959574

[DEV108282C15] Katayama K.-I., Melendez J., Baumann J. M., Leslie J. R., Chauhan B. K., Nemkul N., Lang R. A., Kuan C.-Y., Zheng Y. and Yoshida Y. (2011). Loss of RhoA in neural progenitor cells causes the disruption of adherens junctions and hyperproliferation. *Proc. Natl. Acad. Sci. USA* 108, 7607-7612 10.1073/pnas.1101347108 21502507 PMC3088619

[DEV108282C16] Kim W.-Y., Zhou F.-Q., Zhou J., Yokota Y., Wang Y.-M., Yoshimura T., Kaibuchi K., Woodgett J. R., Anton E. S. and Snider W. D. (2006). Essential roles for GSK-3s and GSK-3-primed substrates in neurotrophin-induced and hippocampal axon growth. *Neuron* 52, 981-996 10.1016/j.neuron.2006.10.031 17178402 PMC4167845

[DEV108282C17] Kim W.-Y., Wang X., Wu Y., Doble B. W., Patel S., Woodgett J. R. and Snider W. D. (2009). GSK-3 is a master regulator of neural progenitor homeostasis. *Nat. Neurosci.* 12, 1390-1397 10.1038/nn.2408 19801986 PMC5328673

[DEV108282C18] Komada M., Saitsu H., Kinboshi M., Miura T., Shiota K. and Ishibashi M. (2008). Hedgehog signaling is involved in development of the neocortex. *Development* 135, 2717-2727 10.1242/dev.015891 18614579

[DEV108282C19] Laplante M. and Sabatini D. M. (2012). mTOR signaling in growth control and disease. *Cell* 149, 274-293 10.1016/j.cell.2012.03.017 22500797 PMC3331679

[DEV108282C20] Lee A. M., Kanter B. R., Wang D., Lim J. P., Zou M. E., Qiu C., McMahon T., Dadgar J., Fischbach-Weiss S. C. and Messing R. O. (2013). Prkcz null mice show normal learning and memory. *Nature* 493, 416-419 10.1038/nature11803 23283171 PMC3548047

[DEV108282C21] Liang H., Hippenmeyer S. and Ghashghaei H. T. (2012). A Nestin-cre transgenic mouse is insufficient for recombination in early embryonic neural progenitors. *Biol. Open* 1, 1200-1203 10.1242/bio.20122287 23259054 PMC3522881

[DEV108282C22] Ling D. S. F., Benardo L. S., Serrano P. A., Blace N., Kelly M. T., Crary J. F. and Sacktor T. C. (2002). Protein kinase Mzeta is necessary and sufficient for LTP maintenance. *Nat. Neurosci.* 5, 295-296 10.1038/nn829 11914719

[DEV108282C23] Ma X. M. and Blenis J. (2009). Molecular mechanisms of mTOR-mediated translational control. *Nat. Rev. Mol. Cell Biol.* 10, 307-318 10.1038/nrm2672 19339977

[DEV108282C24] MacAulay K., Doble B. W., Patel S., Hansotia T., Sinclair E. M., Drucker D. J., Nagy A. and Woodgett J. R. (2007). Glycogen synthase kinase 3alpha-specific regulation of murine hepatic glycogen metabolism. *Cell Metabol.* 6, 329-337 10.1016/j.cmet.2007.08.013 17908561

[DEV108282C25] Maiese K., Chong Z. Z., Shang Y. C. and Wang S. (2013). mTOR: on target for novel therapeutic strategies in the nervous system. *Trends Mol. Med.* 19, 51-60 10.1016/j.molmed.2012.11.001 23265840 PMC3534789

[DEV108282C26] Mak B. C., Takemaru K.-I., Kenerson H. L., Moon R. T. and Yeung R. S. (2003). The tuberin-hamartin complex negatively regulates beta-catenin signaling activity. *J. Biol. Chem.* 278, 5947-5951 10.1074/jbc.C200473200 12511557

[DEV108282C27] Mak B. C., Kenerson H. L., Aicher L. D., Barnes E. A. and Yeung R. S. (2005). Aberrant beta-catenin signaling in tuberous sclerosis. *Am. J. Pathol.* 167, 107-116 10.1016/S0002-9440(10)62958-6 15972957 PMC1603434

[DEV108282C28] Malagelada C., Lopez-Toledano M. A., Willett R. T., Jin Z. H., Shelanski M. L. and Greene L. A. (2011). RTP801/REDD1 regulates the timing of cortical neurogenesis and neuron migration. *J. Neurosci.* 31, 3186-3196 10.1523/JNEUROSCI.4011-10.2011 21368030 PMC3089438

[DEV108282C29] Marin O., Valiente M., Ge X. and Tsai L.-H. (2010). Guiding neuronal cell migrations. *Cold Spring Harb. Perspect. Biol.* 2, a001834 10.1101/cshperspect.a001834 20182622 PMC2828271

[DEV108282C30] Murakami M., Ichisaka T., Maeda M., Oshiro N., Hara K., Edenhofer F., Kiyama H., Yonezawa K. and Yamanaka S. (2004). mTOR is essential for growth and proliferation in early mouse embryos and embryonic stem cells. *Mol. Cell. Biol.* 24, 6710-6718 10.1128/MCB.24.15.6710-6718.2004 15254238 PMC444840

[DEV108282C31] Pastalkova E., Serrano P., Pinkhasova D., Wallace E., Fenton A. A. and Sacktor T. C. (2006). Storage of spatial information by the maintenance mechanism of LTP. *Science* 313, 1141-1144 10.1126/science.1128657 16931766

[DEV108282C32] Patel S., Doble B. W., MacAulay K., Sinclair E. M., Drucker D. J. and Woodgett J. R. (2008). Tissue-specific role of glycogen synthase kinase 3beta in glucose homeostasis and insulin action. *Mol. Cell. Biol.* 28, 6314-6328 10.1128/MCB.00763-08 18694957 PMC2577415

[DEV108282C33] Qu Q., Sun G., Murai K., Ye P., Li W., Asuelime G., Cheung Y.-T. and Shi Y. (2013). Wnt7a regulates multiple steps of neurogenesis. *Mol. Cell. Biol.* 33, 2551-2559 10.1128/MCB.00325-13 23629626 PMC3700117

[DEV108282C34] Rennebeck G., Kleymenova E. V., Anderson R., Yeung R. S., Artzt K. and Walker C. L. (1998). Loss of function of the tuberous sclerosis 2 tumor suppressor gene results in embryonic lethality characterized by disrupted neuroepithelial growth and development. *Proc. Natl. Acad. Sci. USA* 95, 15629-15634 10.1073/pnas.95.26.15629 9861021 PMC28095

[DEV108282C35] Rosner M., Fuchs C., Siegel N., Valli A. and Hengstschlager M. (2009). Functional interaction of mammalian target of rapamycin complexes in regulating mammalian cell size and cell cycle. *Hum. Mol. Genet.* 18, 3298-3310 10.1093/hmg/ddp271 19505958 PMC2722991

[DEV108282C36] Segarra J., Balenci L., Drenth T., Maina F. and Lamballe F. (2006). Combined signaling through ERK, PI3K/AKT, and RAC1/p38 is required for met-triggered cortical neuron migration. *J. Biol. Chem.* 281, 4771-4778 10.1074/jbc.M508298200 16361255

[DEV108282C37] Sessa A., Mao C.-A., Hadjantonakis A.-K., Klein W. H. and Broccoli V. (2008). Tbr2 directs conversion of radial glia into basal precursors and guides neuronal amplification by indirect neurogenesis in the developing neocortex. *Neuron* 60, 56-69 10.1016/j.neuron.2008.09.028 18940588 PMC2887762

[DEV108282C38] Shema R., Sacktor T. C. and Dudai Y. (2007). Rapid erasure of long-term memory associations in the cortex by an inhibitor of PKM zeta. *Science* 317, 951-953 10.1126/science.1144334 17702943

[DEV108282C39] Song J., Salek-Ardakani S., So T. and Croft M. (2007). The kinases aurora B and mTOR regulate the G1-S cell cycle progression of T lymphocytes. *Nat. Immunol.* 8, 64-73 10.1038/ni1413 17128276

[DEV108282C40] Stambolic V. and Woodgett J. R. (1994). Mitogen inactivation of glycogen synthase kinase-3 beta in intact cells via serine 9 phosphorylation. *Biochem. J.* 303, 701-704. 7980435 10.1042/bj3030701 PMC1137602

[DEV108282C41] Tronche F., Kellendonk C., Kretz O., Gass P., Anlag K., Orban P. C., Bock R., Klein R. and Schütz G. (1999). Disruption of the glucocorticoid receptor gene in the nervous system results in reduced anxiety. *Nat. Genet.* 23, 99-103 10.1038/12703 10471508

[DEV108282C42] Tsai V., Parker W. E., Orlova K. A., Baybis M., Chi A. W. S., Berg B. D., Birnbaum J. F., Estevez J., Okochi K., Sarnat H. B. et al. (2014). Fetal brain mTOR signaling activation in tuberous sclerosis complex. *Cereb. Cortex* 24, 315-327 10.1093/cercor/bhs310 23081885 PMC3888364

[DEV108282C43] van Diepen M. T., Parsons M., Downes C. P., Leslie N. R., Hindges R. and Eickholt B. J. (2009). MyosinV controls PTEN function and neuronal cell size. *Nat. Cell Biol.* 11, 1191-1196 10.1038/ncb1961 19767745 PMC2756284

[DEV108282C44] Volk L. J., Bachman J. L., Johnson R., Yu Y. and Huganir R. L. (2013). PKM-zeta is not required for hippocampal synaptic plasticity, learning and memory. *Nature* 493, 420-423 10.1038/nature11802 23283174 PMC3830948

[DEV108282C45] Wanner K., Hipp S., Oelsner M., Ringshausen I., Bogner C., Peschel C. and Decker T. (2006). Mammalian target of rapamycin inhibition induces cell cycle arrest in diffuse large B cell lymphoma (DLBCL) cells and sensitises DLBCL cells to rituximab. *Br. J. Haematol.* 134, 475-484 10.1111/j.1365-2141.2006.06210.x 16856892

[DEV108282C46] Way S. W., McKenna J. III, Mietzsch U., Reith R. M., Wu H. C.-J. and Gambello M. J. (2009). Loss of Tsc2 in radial glia models the brain pathology of tuberous sclerosis complex in the mouse. *Hum. Mol. Genet.* 18, 1252-1265 10.1093/hmg/ddp025 19150975 PMC2655769

[DEV108282C47] Wu S.-X., Goebbels S., Nakamura K., Nakamura K., Kometani K., Minato N., Kaneko T., Nave K.-A. and Tamamaki N. (2005). Pyramidal neurons of upper cortical layers generated by NEX-positive progenitor cells in the subventricular zone. *Proc. Natl. Acad. Sci. USA* 102, 17172-17177 10.1073/pnas.0508560102 16284248 PMC1288007

[DEV108282C48] Zhang D., Contu R., Latronico M. V. G., Zhang J. L., Rizzi R., Catalucci D., Miyamoto S., Huang K., Ceci M., Gu Y. et al. (2010). MTORC1 regulates cardiac function and myocyte survival through 4E-BP1 inhibition in mice. *J. Clin. Invest.* 120, 2805-2816 10.1172/JCI43008 20644257 PMC2912201

