# Supplementary material for: mTOR regulates brain morphogenesis by mediating GSK3 signaling
Source: Development. 2014 Nov;141(21):4076–86. doi: 10.1242/dev.108282 (PMC4302893; doi:10.1242/dev.108282)
Supplement: Supplementary Material [file supp_141_21_4076__index.html]

Supplementary Material 

# mTOR regulates brain morphogenesis by mediating GSK3 signaling

## DEV108282 Supplementary Material

**Files in this Data Supplement:**

- Supplementary Material
