# Supplementary material for: mTOR regulates brain morphogenesis by mediating GSK3 signaling
Source: Development. 2014 Nov;141(21):4076–86. doi: 10.1242/dev.108282 (PMC4302893; doi:10.1242/dev.108282)
Supplement: Supplementary Material [file supp_dev.108282_DEV108282supp.pdf]

Supplemental Figure 1

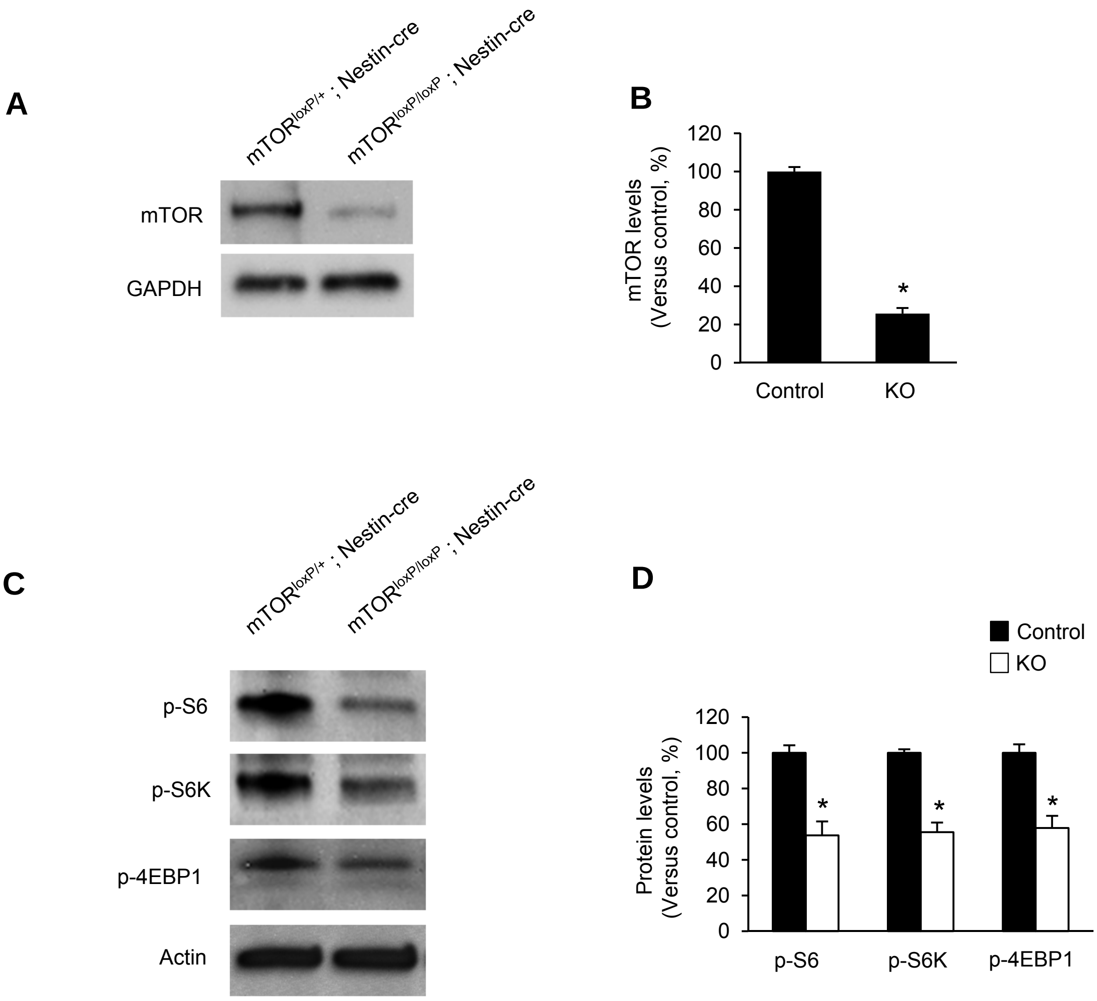

Supplemental Figure 2

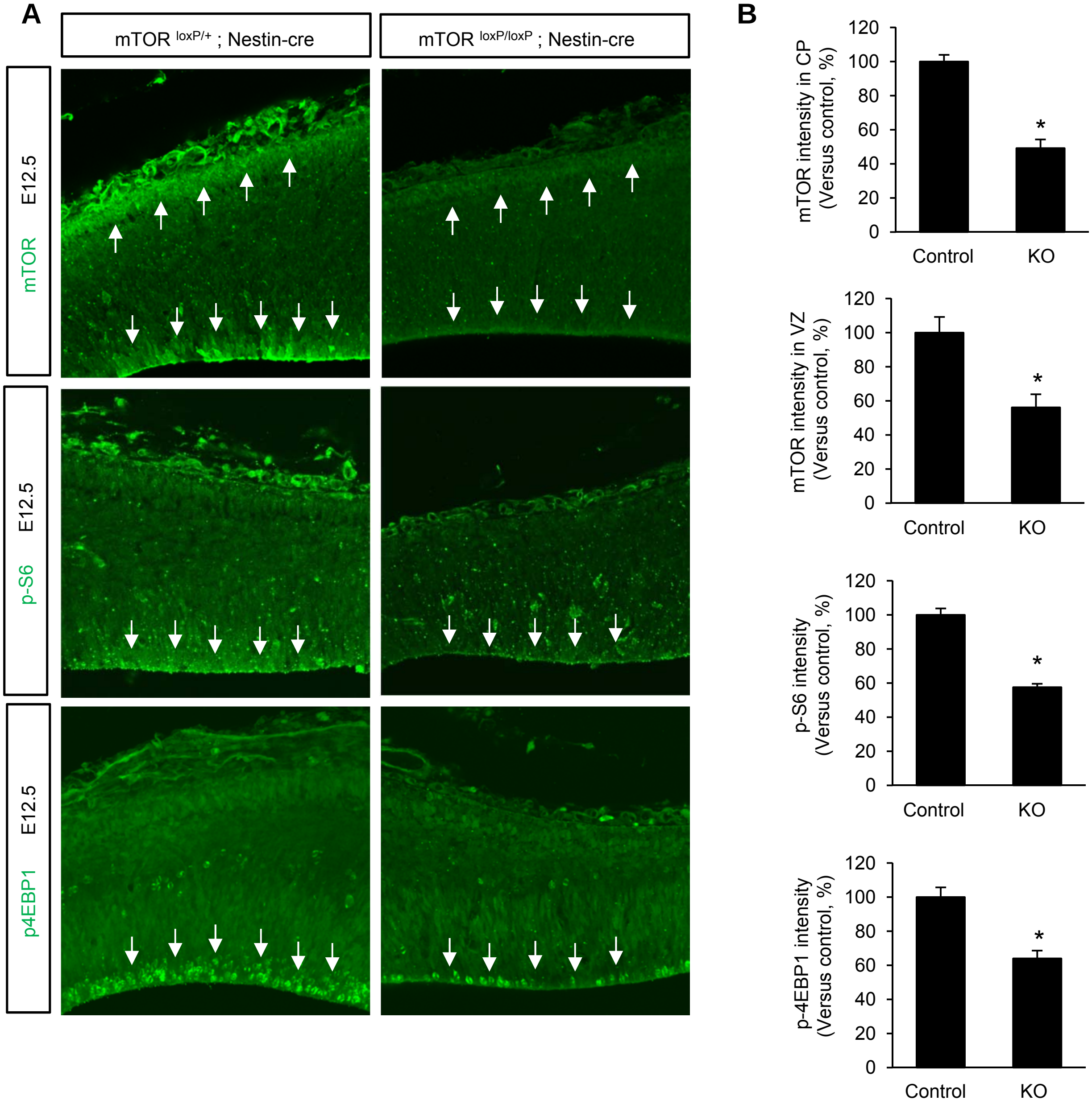

Supplemental Figure 3

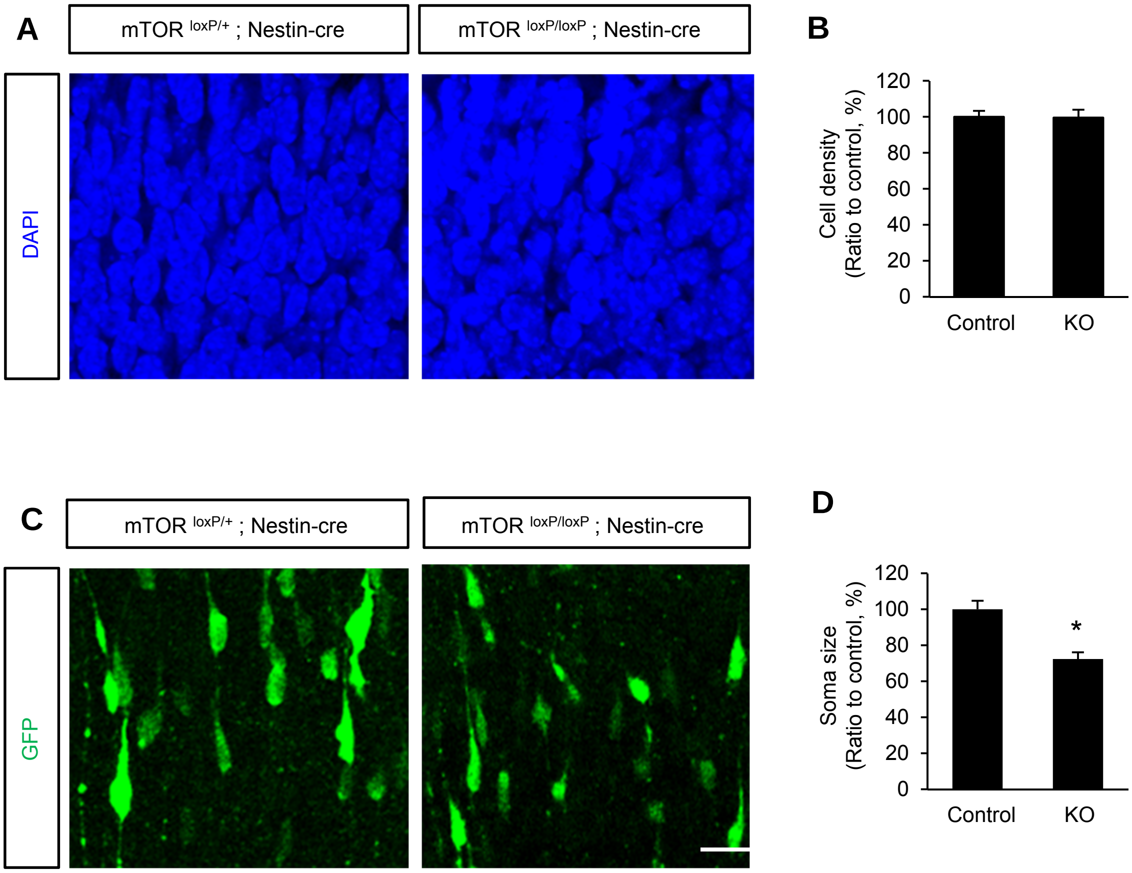

Supplemental Figure 4

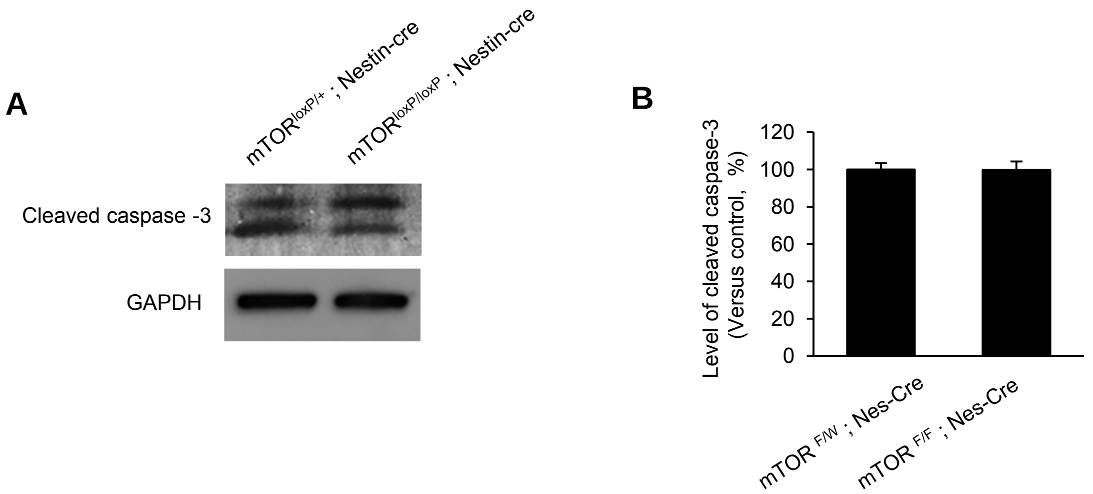

Supplemental Figure 5

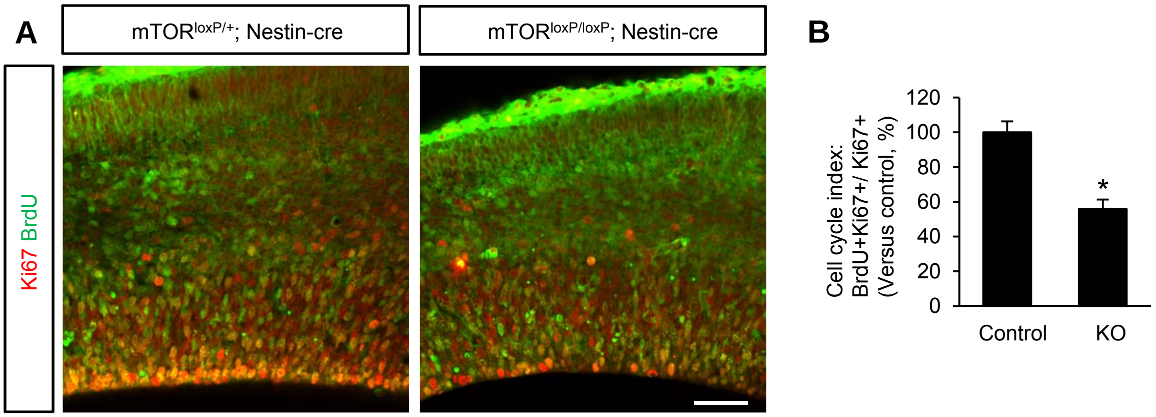

Supplemental Figure 6

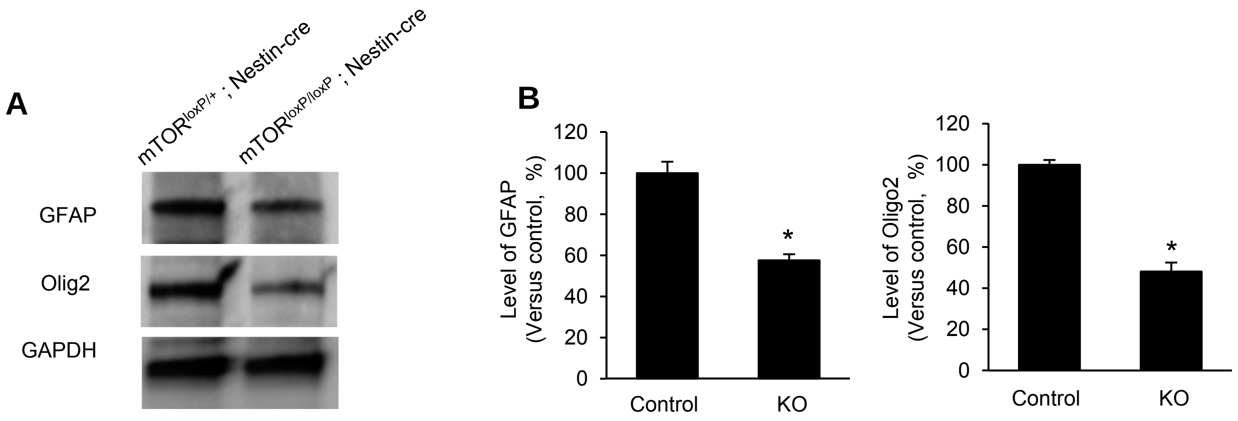

Supplemental Figure 7

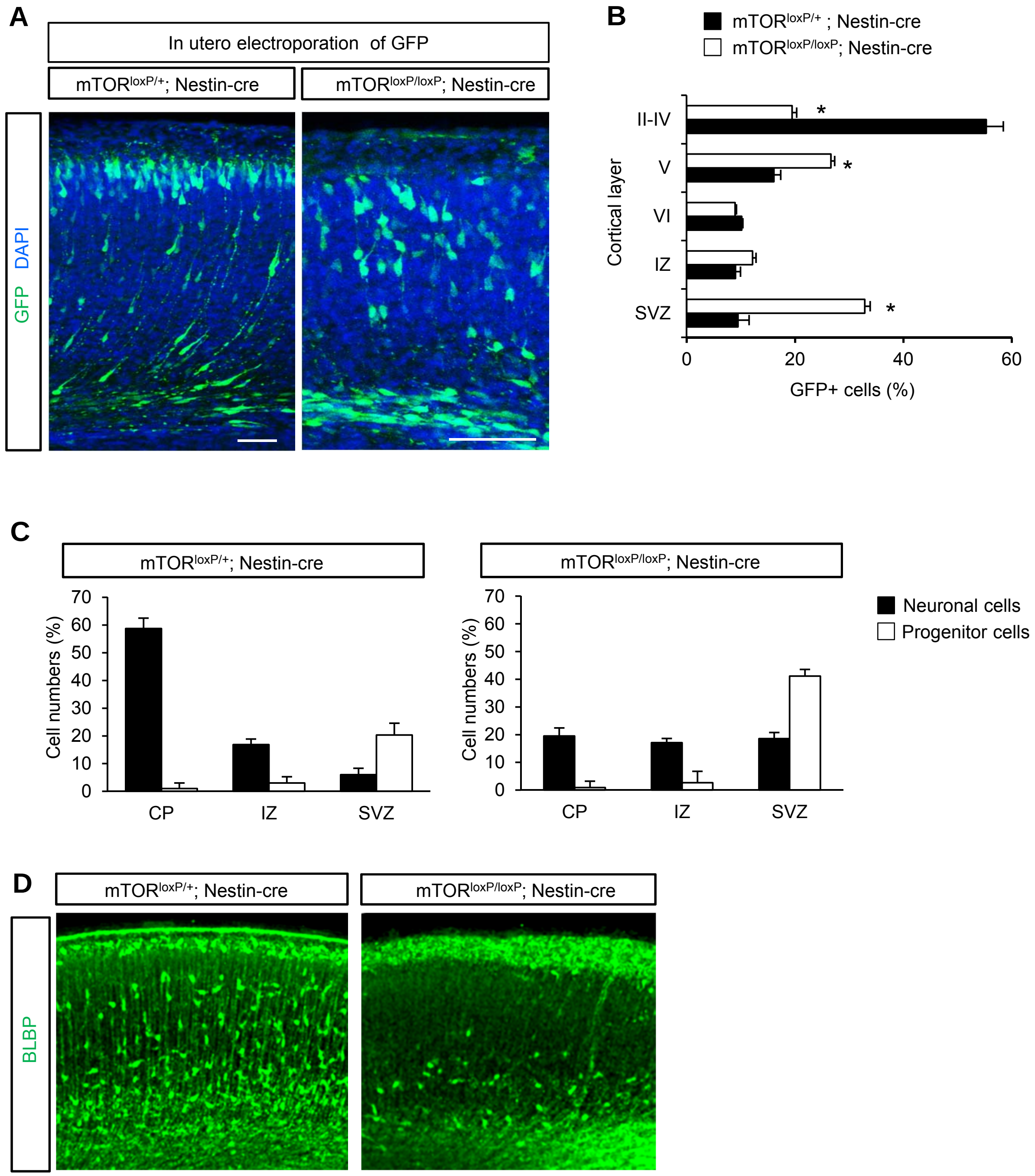

Supplemental Figure 8

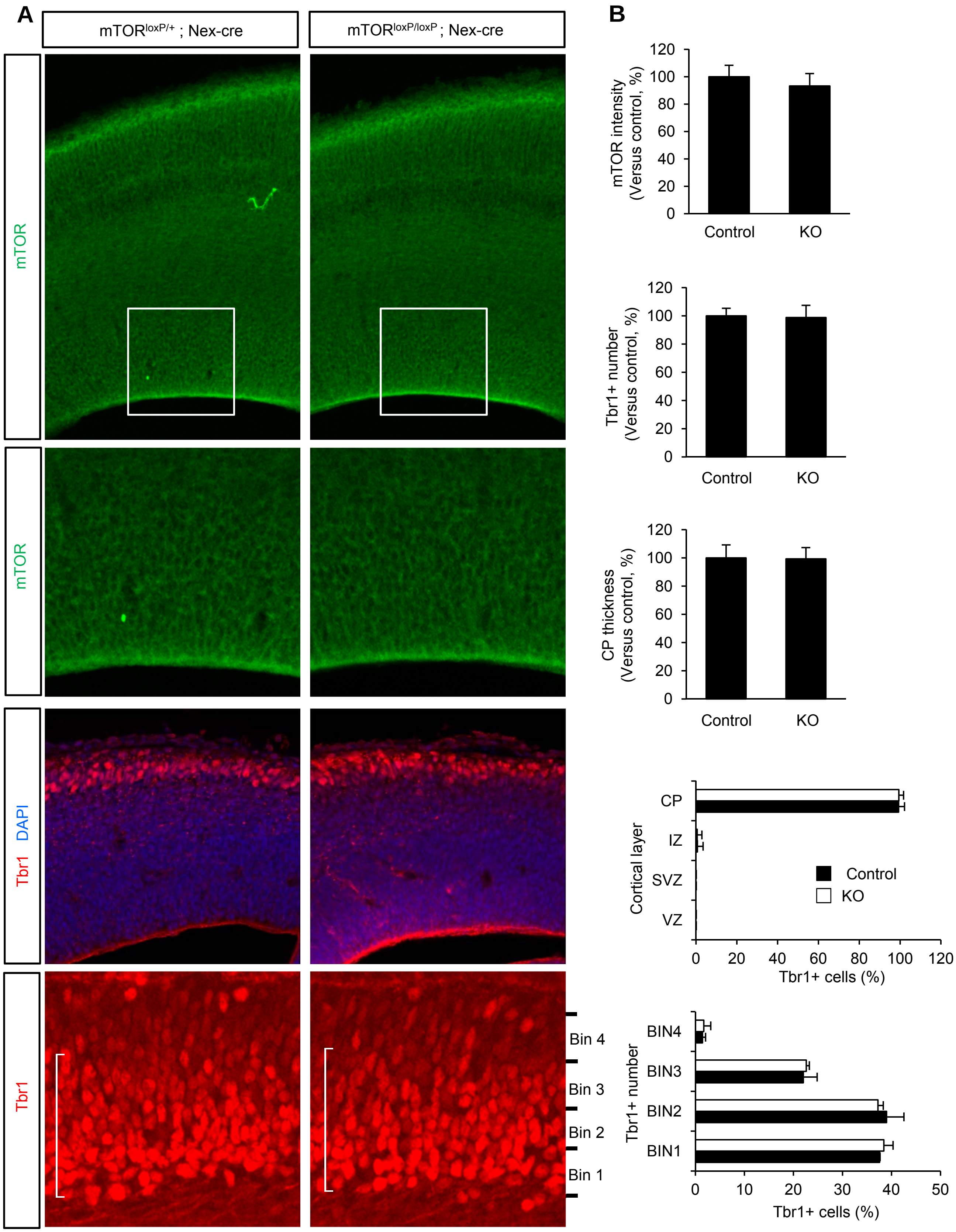

Supplemental Figure 9

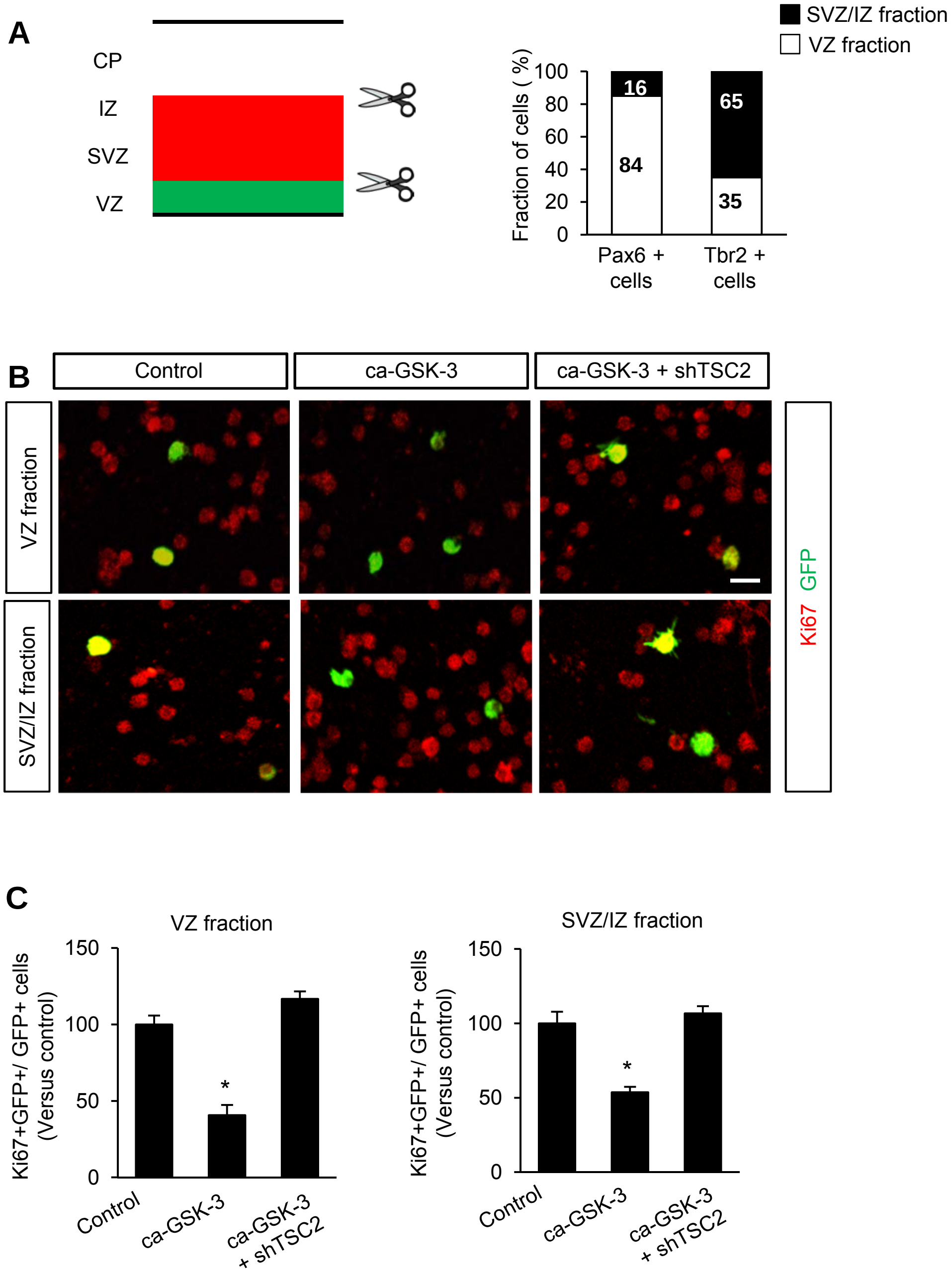

Supplemental Figure legends

Supplemental Figure 1. The levels of mTOR and phosphorylated targets in *mTor$^{loxP/loxP}$; Nestin-cre* mice

(A) Western blot shows mTOR protein is almost completely eliminated in E15.5 brain tissues of *mTor$^{loxP/loxP}$; Nestin-cre* mice. (B) Quantification of (A). The mTOR level was decreased by 76 % in *mTor$^{loxP/loxP}$; Nestin-cre* brains compared to controls. Data were shown as relative changes vs. control. Control: *mTor$^{loxP/+;}$ Nestin-cre*. KO: *mTor$^{loxP/loxP}$; Nestin-cre.* * p<0.05. (C) Phosphorylation of mTOR target proteins is inhibited in *mTor$^{loxP/loxP}$; Nestin-cre* brains. Western blotting using E15.5 brain lysates showed that the levels of the phosphorylated targets were reduced by approximately 50%. (D) Qantification of (C). Control: *mTor$^{loxP/+;}$ Nestin-cre*. KO: *mTor$^{loxP/loxP}$; Nestin-cre.* * p<0.05.

Supplemental Figure 2. mTOR deletion in *mTor$^{loxP/loxP}$; Nestin-cre* mice

(A) mTOR expression in control and *mTor$^{loxP/loxP}$; Nestin-cre* brains. mTOR expression was assessed by immunostaining of E12.5 brain samples. mTOR is expressed in the VZ/SVZ and the cortical plate in control tissues, but the expression was reduced in *mTor$^{loxP/loxP}$; Nestin-cre* brains. Similar patterns were observed in brain samples immunostained with p-4EBP1 and p-S6 antibodies. Arrow indicates the regions that highly express the mTOR components. (B) The fluorescence intensities of mTOR, phospho-S6, and phospho-4EBP1 immunostaining shown in (A) were quantified by ImageJ. Data were presented as relative changes vs. control. Control: *mTor$^{loxP/+;}$ Nestin-cre*. KO: *mTor$^{loxP/loxP}$; Nestin-cre.* * p<0.05.

Supplemental Figure 3. Cell density and size in mTOR-deleted brains

(A) Cell density is not changed in *mTor$^{loxP/loxP}$; Nestin-cre* brains. E15.5 control and mTOR-deleted brains were stained with DAPI. (B) Quantification of (A). There is no significant difference in cell density between control and mTOR-deleted brains. Data were shown as relative changes vs. control. Control:

mTor$^{loxP/+;}$ *Nestin-cre*. KO: *mTor*$^{loxP/loxP}$*; Nestin-cre.* (C) A GFP construct was electroporated in utero into E13.5 control and *mTor*$^{loxP/loxP}$*; Nestin-cre* brains. The sizes of GFP-positive cells were assessed three days later. Scale bar, 30 μm. (D) Quantification of (C). Control: *mTor*$^{loxP/+;}$ *Nestin-cre*. KO: *mTor*$^{loxP/loxP}$*; Nestin-cre.* * $p<0.05$.

Supplemental Figure 4. Levels of cleaved-caspase 3 in *mTor*$^{loxP/loxP}$*; Nestin-cre* mice

(A) The levels of cleaved-caspase 3 were assessed in control and *mTor*$^{loxP/loxP}$*; Nestin-cre* brains by Western blotting. (B) Quantification of (A). No significant changes in cleaved caspase-3 levels were found between controls and *mTor*$^{loxP/loxP}$*; Nestin-cre* brain samples. Data were shown as relative changes vs. control. Control: *mTor*$^{loxP/+;}$ *Nestin-cre*. KO: *mTor*$^{loxP/loxP}$*; Nestin-cre.*

Supplemental Figure 5. mTOR-deleted neural porgenitors exhibit lengthened cell cycle

(A) To analyze cell cycle length, E14.5 control and *mTor*$^{loxP/loxP}$*; Nestin-cre* mice were pulse-labeled with BrdU for 1 hour and then brains were collected for immunostaining with BrdU (green) and Ki67 (red) antibodies. Scale bar, 50 μm. (B) Quantification of cell cycle index. The labeling index of cell cycle length is defined as the fraction of BrdU- and Ki67-positive cells in total Ki67-positive cells in the brain. The results were shown as relative changes vs. control. The decreased labeling index suggests prolonged cell cycles in mTOR-deficient neural progenitors. Control: *mTor*$^{loxP/+;}$ *Nestin-cre*. KO: *mTor*$^{loxP/loxP}$*; Nestin-cre.* * $p<0.05$.

Supplemental Figure 6. Levels of GFAP and Olig2 in *mTor*$^{loxP/loxP}$*; Nestin-cre* mice

(A) Using E15.5 control and *mTor*$^{loxP/loxP}$*; Nestin-cre* brain lysates, Western blotting was performed with GFAP and Olig2 antibodies. (B) Quantification of (A). The levels of GFAP and Olig2 were significantly decreased in *mTor*$^{loxP/loxP}$*; Nestin-cre* brains compared to control tissues. Data were shown as relative changes vs. control. Control: *mTor*$^{loxP/+;}$ *Nestin-cre*. KO: *mTor*$^{loxP/loxP}$*; Nestin-cre.* * $p<0.05$.

Supplemental Figure 7. Neuronal placement in *mTor*$^{F/F}$*; Nestin-cre* mice

(A) E13.5 control and *mTor*$^{loxP/loxP}$*; Nestin-cre* mice were electroporated in utero with a GFP plasmid. The brain samples of the mice were collected at E18 and then cortical placement of GFP-positive cells were visualized. Scale bar, 50 μm. (B) Quantification of GFP-positive cells in the cerebral cortex. While GFP-positive cells in control brain were placed mostly in cortical plate, GFP cells in *mTor*$^{F/F}$*; Nestin-cre* brains were found throughout the cerebral cortex. Control: *mTor*$^{loxP/+;}$ *Nestin-cre*. KO: *mTor*$^{loxP/loxP}$*; Nestin-cre.* * $p<0.05$. (C) Quantification of GFP-positive cell types after in utero electroporation of a GFP construct as described in (A). Control and *mTor*$^{loxP/loxP}$*; Nestin-cre* brains were co-immunostained with GFP and MAP2 or GFP and Pax6 (or Tbr2). Most GFP-positive cells were MAP2-positive neurons, while GFP-positive cells in the VZ/SVZ were divided to MAP2-positive neurons and PAX6-positive (orTbr2-positive) progenitors. Control: *mTor*$^{loxP/+;}$ *Nestin-cre*. KO: *mTor*$^{loxP/loxP}$*; Nestin-cre.* * $p<0.05$. (D) Control and *mTor*$^{loxP/loxP}$*; Nestin-cre* brains were immunostained with a BLBP antibody. Radial glial fibers were formed normally in control tissues. However, the glial fibers were disrupted in *mTor*$^{loxP/loxP}$*; Nestin-cre* brain samples.

Supplemental Figure 8. *mTor*$^{loxP/loxP}$*; Nex-cre* mice at E14.5

(A) Effects of mTOR deletion using Nex-cre mice at early developmental stage, E14.5. Top panels: mTOR immunostaining in the cerebral cortex. mTOR expression was examined by immunostaining of E14.5 control and *mTor*$^{loxP/loxP}$*; Nex-cre* brain samples. Second row panels: Higher magnification images of white square areas in top panels. Third row panels: Brain sections from E14.5 control (*mTor*$^{loxP/+}$*; Nex-cre*) and *mTor*$^{loxP/loxP}$*; Nex-cre* mice were immunostained with Tbr1 antibody. Cells were counterstained by DAPI. Bottom panels: Higher magnification images of Tbr1-immunostanined samples. (B) Quantification of (A). *mTor*$^{loxP/loxP}$*; Nex-cre* mice did not show altered positioning, number, and layer thickness of Tbr1-positive cells, compared to controls. The fluorescence intensities of mTOR were

quantified by ImageJ. Data were presented as relative changes vs. control. Control: *mTor*$^{loxP/+;}$ *Nex-cre*. KO: *mTor*$^{loxP/loxP}$*; Nex-cre*. * p<0.05.

Supplemental Figure 9. TSC2 knockdown suppresses the inhibitory effects of GSK-3 on Pax6-positive or Tbr2-positive progenitor proliferation

(A) Left panel: A diagram showing embryonic cerebral cortex. Tissues containing the ventricular zone (green) or the subventricular/intermediate zone (red) from E13.5 mouse brains were fractionated by brain slicing and micro-dissection. Pax6-positive progenitors are mostly distributed in the ventricular zone while a major portion of Tbr2-positive cells are localized within the subventricular zone and intermediate zones (Arai et al., 2011). Right panel: Percentages of Pax6-positive and Tbr2-positive cells in the VZ fraction and the SVZ/IZ fraction were quantified. Most Pax6-positive cells were found in the VZ fraction. A major population of Tbr2-positive cells was seen in the SVZ/IZ fraction. (B) TSC2 knockdown suppresses the inhibitory effects of GSK-3 on proliferation of PAX6-postive and Tbr2-positive cells. E13.5 neural progenitors from each fraction were cultured and transfected with control GFP, ca-GSK-3-GFP, or ca-GSK-3-GFP and shTSC2. Then, proliferating neural progenitors were assessed by Ki67 and GFP co-immunostaining. (C) The effects of GSK-3, shTSC2, and shGSK-3 on cell proliferation were assessed by quantifying the Ki67 and GFP double-positive cells in total GFP-positive cells. The results were shown as relative changes vs. control. * p<0.05.
